# Microbial named entity recognition and normalisation for AI-assisted literature review and meta-analysis

**DOI:** 10.1093/bioinformatics/btag418

**Published:** 2026-06-20

**Authors:** Dhylan Patel, Antoine D Lain, Avish Vijayaraghavan, Nazanin Faghih-Mirzaei, Monica N Mweetwa, Meiqi Wang, Tim Beck, Joram M Posma

**Affiliations:** Section of Bioinformatics, Division of Systems Medicine, Department of Metabolism, Digestion and Reproduction, Imperial College London, London W12 0NN, United Kingdom; Department of Life Sciences, Imperial College London, London SW7 2AZ, United Kingdom; Section of Bioinformatics, Division of Systems Medicine, Department of Metabolism, Digestion and Reproduction, Imperial College London, London W12 0NN, United Kingdom; Section of Bioinformatics, Division of Systems Medicine, Department of Metabolism, Digestion and Reproduction, Imperial College London, London W12 0NN, United Kingdom; UKRI Centre for Doctoral Training in AI for Healthcare, Department of Computing, Imperial College London, London SW7 2AZ, United Kingdom; Section of Bioinformatics, Division of Systems Medicine, Department of Metabolism, Digestion and Reproduction, Imperial College London, London W12 0NN, United Kingdom; Section of Bioinformatics, Division of Systems Medicine, Department of Metabolism, Digestion and Reproduction, Imperial College London, London W12 0NN, United Kingdom; Tropical Gastroenterology and Nutrition Group (TROPGAN), School of Medicine, University of Lusaka, Lusaka, Zambia; Section of Bioinformatics, Division of Systems Medicine, Department of Metabolism, Digestion and Reproduction, Imperial College London, London W12 0NN, United Kingdom; Centre for Health Informatics, School of Medicine, University of Nottingham, Nottingham NG7 2RD, United Kingdom; Health Data Research (HDR) UK, London NW1 2BE, United Kingdom; Section of Bioinformatics, Division of Systems Medicine, Department of Metabolism, Digestion and Reproduction, Imperial College London, London W12 0NN, United Kingdom; Health Data Research (HDR) UK, London NW1 2BE, United Kingdom

## Abstract

**Motivation:**

Manual curation of biomedical literature is slow and error-prone and while large language models trained on general texts have shown to be useful for text summarisation, these methods lack the domain-specific expertise required to perform this task accurately. Here we describe the creation of the first microbiome-specific text corpus, use this to train deep learning algorithms for named-entity recognition (NER) and entity linking (EL), and demonstrate their use to meta-analyse microbiome literature.

**Results:**

The training and validation set (*n = *1410) contained a total of 90 150 annotations (both long form and abbreviations). Using the gold-standard test set (*n = *288), with an inter-annotator agreement rate of 99.52% for NER and 88.31% for EL, the trained models were evaluated and our fine-tuned BioBERT model achieved an F1-score of 96% for NER surpassing a rule- and dictionary-based annotation pipeline (94%). For EL the accuracy obtained by the deep learning models greatly surpassed that of the pipeline (91% vs 69%). Evaluated across the entire available literature (*n = *6927) across 14 domains, our models annotate an entire full-text document in only 7 seconds.

**Availability:**

All codes are available for automatic annotation and model training, with instructions on how to deploy the model on new text, from GitHub at https://github.com/omicsNLP/microbELP and Zenodo at https://dx.doi.org/10.5281/zenodo.20613467. The redistributable, annotated training set and unannotated test set are made available from Zenodo at https://dx.doi.org/10.5281/zenodo.17305410 with the redistributable, human-labelled test set hosted as benchmark on Codabench at https://www.codabench.org/competitions/10913/ (for NER only) and at https://www.codabench.org/competitions/11581/ (for NER+EL) for evaluation. The annotated documents for all available literature are hosted separately on Zenodo at https://dx.doi.org/10.5281/zenodo.17288826.

## 1 Introduction

Manual extraction of information from large numbers of publications is time-consuming, labour-intensive and may lead to human errors. With the biomedical literature expanding at an increased rate (Bornmann and Mutz 2015), there is a clear need to develop new methods for specialised tasks; tasks that large language models (LLMs), such as GPT (Radford *et al.* 2018), usually lack extensive training data for, given these LLMs are often pre-trained on general texts and therefore lack specific biomedical knowledge to excel in these areas. It has been demonstrated by different surveys that ‘small’ language models (LMs) such as BERT-based models (Alsentzer *et al.* 2019, Beltagy *et al.* 2019, Gu *et al.* 2022, Lee *et al.* 2020, Yasunaga *et al.* 2022) outperform LLMs for named entity recognition (NER) (Chen *et al.* 2023, Hu *et al.* 2024, Jahan *et al.* 2024), entity linking (EL, also known as named entity normalisation) (Jahan *et al.* 2024), text classification (Chen *et al.* 2023, Jahan *et al.* 2024) and other tasks not relying on text generation. While LLMs generally perform better for question-answering and relation extraction tasks (Chen *et al.* 2023, Jahan *et al.* 2024).

To use any type of LM (small or large) for literature review, several elements determine their performance, and therefore usefulness. Domain specificity is important as there may be specific vocabularies and terminologies used, and nuances made in (biomedical) scientific literature that are less common in general text. Training or fine-tuning existing models for specific tasks require structured text corpora with enough annotated training examples for the task to allow them to pick up relevant language patterns. Some corpora exist in which organisms are annotated, such as CRAFT (Bada *et al.* 2012) (97 full-texts, 7449 organisms), Linnaeus (Gerner *et al.* 2010) (100 full-texts, 4259 organisms), species-800 (Pafilis *et al.* 2013) (800 abstracts, 3708 organisms), BioNLP-ID (Pyysalo *et al.* 2011) (30 full-texts, 3471 organisms), miRNA corpus (Bagewadi *et al.* 2014) (201 abstracts, 546 organisms), CellFinder (Neves *et al.* 2012) (10 full-texts, 438 organisms), and CoDiet (Lain *et al.* 2025) (450 full-texts, 28 443 organisms). Some of these corpora have been used to train LMs that have shown state-of-the-art performance for biomedical NER, such as BioBERT (Lee *et al.* 2020), BERN2 (Sung *et al.* 2022), and SciBERT (Beltagy *et al.* 2019).

The microbiome is the collection of microorganisms that play crucial roles in biochemical reactions by modulating processes essential for maintaining host health. While these are organisms, the problem of using existing organism recognition models relates both to specificity and size, since not all species included are microbes and only a subset of microbes are included in these corpora. Therefore these corpora, and by extension the models trained on them, are not suitable for microbiome NER as these will give rise to both high false positives rates when aiming to target microbiome entities using the organism tag, and high false negative rates as many species are unseen by the training data.

Here, we aim to fill this gap by describing the creation of a microbiome-specific training corpus, a dictionary-based annotation and normalisation workflow (with microbiome-specific rules), construction of an annotated microbiome (bacteria, archaea, fungi) corpus of full-text publications in machine-readable format, and finally contributing four transformer-based LMs trained on these data and evaluated on a manually annotated test set, including two models for NER and two for EL.

## 2 Materials and methods

### 2.1 Data—training corpus

PubMed Central was searched on 14 April 2020 to generate an initial corpus spanning 5 different microbiome domains (airway (from oral and nasal to lung), faecal (stool), skin (skin tissue), urinary (excluding vaginal) and vaginal (vagina, cervix, placenta)) in humans for model training (see below). The search query used is given in the Supplementary Methods, available as supplementary data at *Bioinformatics* online.

The publications were converted from HTML and XML documents, standardised and converted to the machine-readable BioC-JSON format using Auto-CORPus (Beck *et al.* 2022). First, Auto-CORPus converts the main text of each publication from HTML or XML to BioC JSON format. In this file, the pipeline splits the publications into different sections using the Information Artifact Ontology (IAO) (Ceusters 2012) and in each section, each paragraph forms a single ‘text’-feature. Second, Auto-CORPus transforms tables inside publications to a table-JSON format, and it extracts abbreviations from both the main text and from separate abbreviations sections (if available) within the main text and generates another JSON file for abbreviations with linked full definitions. Here, we use the full-text BioC-JSON files for pre-processing, see more details in Wang *et al.* 2024, and to build a silver-standard annotated corpus for DL models.

### 2.2 Data—NCBI taxonomy

The NCBI taxonomy data was downloaded on 1 November 2021 as separate memory dump (dmp) files. All names (scientific, common, blast, other) were extracted including the child and parent identifiers of each entry. The data was filtered for any node that is an ancestor of either txid2 (bacteria), txid2157 (archaea) or txid4751 (fungi). Several data cleaning steps were performed (see Supplementary Methods for details, available as supplementary data at *Bioinformatics* online). The final dictionary was saved as a JSON file containing fields original name, cleaned name, taxonomic rank, taxonomy ID, parent ID and kingdom ID and used subsequently for the annotation pipeline. The summary statistics can be found in Table 1.

**Table 1:** Number of unique identifiers (with number of unique names in brackets) in each of the three microbial kingdoms following data curation.

Rank	Archaea	Bacteria	Fungi
(Super/sub) kingdom	1 (10)	1 (8)	2 (3)
(Sub) phylum	21 (36)	147 (216)	24 (44)
(Sub) class	20 (28)	107 (145)	82 (109)
(Sub) order	29 (33)	253 (309)	254 (314)
(Super/sub) family	49 (54)	627 (702)	873 (980)
Tribe	0 (0)	2 (2)	3 (3)
(Sub) genus	199 (216)	4 254 (4720)	7 058 (7 262)
Section	0 (0)	0 (0)	23 (23)
Species*	901 (1123)	25 653 (33 493)	53 483 (75 223)
Forma	0 (0)	1 (1)	15 (15)
Clade	6 (7)	14 (14)	4 (4)
total	1 226 (1 507)	31 059 (39 610)	61 821 (83 980)

* Includes subspecies and lower insofar these could be mapped to a unique name.

### 2.3 Training and test splits

A total of 1410 documents were used for training our LMs, with another 288 used for testing both the pipeline and the LMs. The document numbers were derived by splitting roughly 80:20 (train: test) across the 5 domains of articles that were targeted: airway (oral to lung), faeces (gut, stool), skin, urinary and vaginal microbiomes. A subset of documents in the training portion of the corpus were used to develop the rules implemented in the annotation pipeline (Supplementary Materials, available as supplementary data at *Bioinformatics* online). The annotated training set (see details on annotations in Table 2) was used as input to the deep learning NER models (below) and split into training and validation portions with a 70:30 ratio within the algorithms to fine-tune the models.

**Table 2:** Characteristics of the silver-annotated training+validation corpus.

Descriptor	Train+Val	Airway*	Faecal*	Skin*	Urinary*	Vaginal*
# of full-text articles	1 410	525	700	142	53	124
Total # annotations in full-text	90 150	35 342	41 570	8 761	3 303	8 273
# of identifiers assigned	93 016	36 593	42 858	9 132	3 397	8 415
# of archaeal annotations	372	92	169	104	3	12
# of bacterial annotations	89 007	34 769	41 290	8 695	3350	8 123
# of fungal annotations	3 541	1 701	1 351	318	41	278
# of annotations with 5+ identifiers	96	31	48	15	3	2
# of abbreviated entities	17 361	7 379	6 770	1 803	441	2 113
Median (IQR) # annotations per article	57 (31–91)	61 (37–93)	51 (27–86)	56 (31–88)	57 (19–102)	57 (29–92)

The number of identifiers exceeds the number of annotations as some annotations cannot be resolved to a single identifier due to ambiguity. (*articles can be assigned to multiple domains).

Each full-text article from the test set (*n = *288) was converted to BioC XML and uploaded to a local installation of TeamTat (Islamaj *et al.* 2020) and annotated by two independent annotators resulting in 23 561 microbiome entities and their corresponding identifiers. Our annotators had an inter-annotator agreement of 88.97% F1-score. Disagreements between pairs of annotators were resolved by a third annotator to arbitrate. All disagreements were resolved through this process, resulting in a minimum of two human annotators having verified each annotation.

### 2.4 Deep learning models for NER

We used the 1410 files obtained from our search strategy, already converted from HTML and XML formats to BioC JSON, and ran our rule-based annotation pipeline (see Supplementary Methods, available as supplementary data at *Bioinformatics* online) to generate annotations. The annotated BioC JSON files were then converted to the BIO2 (Ramshaw and Marcus 1995) format using bins of a maximum size of 512 tokens, stopping at the end of the most recently completed sentence. During this conversion, we excluded the reference sections to reduce potential noise during training due to the formatting of references being different from normal sentences in full text. After conversion to BIO2, data was split into training and validation, ensuring the same distribution of the five broad domains used in our search strategy.

We selected two pre-trained models, BioBERT (*biobert-base-cased-v1.2*) and SciBERT (*scibert_scivocab_cased*), to fine-tune on our data. For each model, we repeated the process using nine different random seeds to evaluate performance across varying weight initialisations and reduce the influence of luck. An odd number of models was chosen to allow selecting the median model for EL (see Evaluation Metrics below). We did not perform any hyperparameter search but fine-tuned each model with a fixed set of parameters based on prior work (Beltagy *et al.* 2019, Lee *et al.* 2020): a batch size of 24 bins, a learning rate of $3\times 10^{-5}$, five epochs, and a weight decay of $1\times 10^{-5}$. The LLaMa-3.1-8B and gpt-oss-20b models were used with few-shot prompting (see GitHub for prompts) for comparison.

All models were trained on a Linux workstation with an Intel 24-core i9-13900K CPU with 192GB RAM (4 × 48GB) and an Nvidia GeForce RTX 4090 GPU (24GB memory, 16 384 CUDA cores). The epoch with the highest F1 score (see below) for the validation dataset was stored and used to evaluate on the manually annotated test set. Training took 13 minutes per model on average.

#### 2.4.1 Deep learning models for EL

To train our deep learning model for EL, we used the same training and validation sets as those used for NER. The most common approach to training entity representation models involves constructing a pair-wise training dataset. Accordingly, we extracted both the entity and the identifier found by the pipeline when creating the training and validation datasets. We chose to retrain the BioSyn architecture (Sung *et al.* 2020) due to its use of embedding space information, its capacity to handle out-of-vocabulary terms, and its ability to perform synonym marginalisation. Similar to the NER task, reference sections were excluded from the datasets. To retrain a BioSyn model, a dictionary is required on top of the datasets. For this purpose, we used the file generated in the ‘Data—NCBI Taxonomy’ section as the dictionary for training, validation, and testing. Further details regarding the input formatting can be found in the original GitHub repository (https://github.com/dmis-lab/BioSyn).

We used five pre-trained models to develop our BioSyn models. Three models were fine-tuned using their original weights: BioBERT (Lee *et al.* 2020), SciBERT (Beltagy *et al.* 2019), and SapBERT (Liu *et al.* 2021). The remaining two models were derived from the previous section: the fifth-best (median) fine-tuned BioBERT model and the median fine-tuned SciBERT model, as determined by their performance on the validation set. We did not conduct hyperparameter fine-tuning but instead employed the following fixed parameter set: top-k of 20, 10 epochs, batch size of 16, learning rate of $1\times 10^{-5}$, hybrid scoring, and a dense ratio of 0.5. All models were trained on the same Linux workstation as described previously, with an average training time of 40 minutes per model.

#### 2.4.2 Comparison of our models for species recognition using BERN2

We compare our microbiome NER results with the BERN2 multi-task model (Sung *et al.* 2022), which is based on BioBERT, for the species entity tag. For a faster inference on our large corpus, we followed the authors’ guidelines in the BERN2 repository (https://github.com/dmis-lab/BERN2) to install and run a local version of the model on the same Linux workstation as above. Once the BERN2 model is running on a local port within its conda environment, we make requests to it using the URL associated with the port. Running our corpus through the model returns a set of found entities per paper (and optionally per paragraph) which fall into one of nine biomedical classes: gene/protein, disease, drug/chemical, species, mutation, cell line, cell type, DNA, and RNA. We filter entities for the species class to enable microbiome NER and EL comparison to our models.

#### 2.4.3 Evaluation metrics

To evaluate the performance of the annotation pipeline and DL models for NER, we calculate 3 properties of a confusion matrix: true positive (TP) entities, false positives (FPs) and false negatives (FNs). FPs are entities predicted to be a microbial entity by a model which does not match an entity in the test set. FNs are entities that were not annotated by the model but are contained in the manually annotated test set. Using these measures, we calculate the precision ($\frac{TP}{TP+FP}$), recall ($\frac{TP}{TP+FN}$) and F1-score ($\frac{2\times TP}{2\times TP+FP+FN}$).

We calculate these metrics according to three methods of evaluating an entity: strict, relaxed and proportional overlap (token based). Strict indicates the exact span of an entity must be found to be counted as TP. A TP for relaxed is counted when the span includes at least one character from the annotation. We define the proportional overlap measure to be an intermediate of the two prior ones, for each annotation the number of tokens correctly identified (proportional to the length of the found entity) is counted towards TP, the proportion of missed tokens to FN and any additional tokens proportionally to FP. The proportional overlap does not penalise partial positive matches, i.e. where only the genus is correctly identified (when part of a species name).

We evaluate the performance of the annotation pipeline and DL models for EL using two accuracy metrics: Acc@1 and Acc@5, focussing on the model’s ability to rank the correct identifier among the top 1 and 5, respectively, candidates predicted for each entity. Acc@1 measures the proportion of cases where the top-ranked candidate matches the correct identifier as defined in Equation 1 where *N* is the total number of entities, $y_{i}$ the correct identifier for the *i*-th entity, $x_{i}$ the input entity representation, and ⊮{·} an indicator function that returns 1 if the condition inside is true, and 0 otherwise. $\text{Top}1(x_{i})$ represents the highest-ranked candidate for $x_{i}$. Acc@5 extends this to the top five ranked candidates.


(1) $$\text{Acc}@1=\frac{1}{N}\sum_{i=1}^{N}⊮\{y_{i}\in \text{Top}1(x_{i})\}$$

### 2.5 AI-assisted literature summarisation

The best performing model was applied to all machine-accessible Open Access documents from 14 microbiome domains that were published with Open Access from 1 January 2000 to 31 December 2023: oral (mouth and saliva), nasal (including nasopharynx), pharynx (throat), lung (lung, respiratory tract), upper digestive tract (oesophagus to duodenum), lower digestive tract (jejunum to anus), faecal (faecal, stool), hepatobiliary (liver, pancreas, bile duct, gall bladder), skin (skin, toe, foot, elbow fold, forehead), female reproductive (vagina, cervix, endometrium, but not urinary), placental (placenta), breast milk (human milk, colostrum, lactation), male reproductive (testicle, semen, but not urinary), and urinary (urine but not vaginal or testicular) microbiomes. A total of 8120 articles were returned from the search, with a total of 6927 available from PubMed Central’s Open Access corpus and/or Elsevier’s API that were extracted using CADMUS (Campbell *et al.* 2026) and converted to BioC-JSON files with Auto-CORPus (Beck *et al.* 2022). We extracted the annotated entities from the results sections (IAO : 0000318) and a list of each unique taxonomic identifier in these sections was extracted for each article. These lists were then aggregated per microbiome domain resulting in a list of identifiers and the number of times articles in that domain have reported them.

#### 2.5.1 Taxonomic tree

A combined set of all taxonomic identifiers found in any section of any document across the 14 domains was compiled and used to create a ‘reference’ taxonomic tree. We compare the counts of the domain specific trees with the reference tree through a resampling approach (see Supplementary Methods for details, available as supplementary data at *Bioinformatics* online) to generate random trees with the same degree structure as the domain tree and calculate an empirical *P*value, adjusted for multiple testing using the False Discovery Rate (FDR, Q-value), to estimate the proportion that random sampling results in higher counts than the domain tree. The Q-values are visualised on a −log10 scale, with significance indicated by Q < 0.05 (or 1.30 on the −log10 scale). For any node in the graph, a significant Q-value indicates that this microbe is mentioned more than would be expected by random chance.

### 2.6 Code and data availability

NCBI Taxonomy data is available from the NCBI FTP server. The annotation pipeline code, code to train and inference NER, and code to inference EL is available from GitHub. The fine-tuned models are available from HuggingFace for both NER and EL. The OA (licences permitting redistribution) training set for NER (with annotations) and test set (without annotations) are available from Zenodo. The OA test set (with annotations) is hosted on Codabench to allow submissions to benchmark against the gold standard test set for NER only, as well as NER + EL. The list of identifiers in the reference tree and each domain tree is available through a separate Zenodo repository, where also the automatically annotated documents for each domain can be found. Code to map results from a single study to an existing tree graph (reference or domain specific) can be found on GitHub as a stand-alone function. All redistributed documents permitted redistribution at the time of downloading these documents; their main text remains the intellectual property and views of the original authors.

## 3 Results

### 3.1 Taxonomy dictionary and corpus creation

We curated a dictionary containing only the microbial (archaea, bacteria, fungi) entities from the NCBI Taxonomy database consisting of a total of 125 097 unique names mapping to 94 106 unique identifiers (Table 1), from (super)kingdom to species level. This dictionary was used as input to our annotation pipeline to automatically annotate 1410 full-text articles for training and validation of deep learning models. A total of 90 150 annotations were made across the documents, with a median number of annotations between 51 and 61 for each of the five categories (Table 2). The majority of entities in the dictionary are fungi, however most annotations in the full-text documents are bacteria.

### 3.2 Model evaluation for entity recognition

We tested the models on a gold-standard test set of 288 documents that were annotated by two independent human researchers, with disagreements settled by a third arbiter. The inter-annotator agreement (across 20 486 annotations), represented by the F1-score, was 99.52% for NER. The pipeline without applying normalisation (i.e. exact matching of the recognised entity to an entry in the dictionary to filter out false positives) achieved a strict F1-score of 86.49% on the test set, and a relaxed F1 of 91.90% (see Table 3). Adding normalisation into the pipeline increases the performance to a strict F1 of 93.08% and relaxed F1 of 93.87%.

**Table 3:** Performance metrics for different models (name and yes/no normalisation as part of entity recognition) evaluated on the manually annotated test set (*n = *288 full text articles, total of 20 486 annotations) for entity recognition.

Model	Norm	Strict		
		Precision	Recall	F1-score
**Pipeline**	No	89.42	83.74	86.49
**Pipeline**	Yes	**99.07**	87.78	93.08
**BioBERT**	No	97.99 ± 0.68	**91.11** ± 0.99	94.42 ± 0.81
**SciBERT**	No	98.43 ± 0.30	90.74 ± 0.22	**94.43** ± 0.12
**LLaMa-3.1-8B**	No	61.41	35.36	44.88
**gpt-oss-20b**	No	89.73	82.74	86.09
**BERN2**	Yes	39.10	23.17	29.10

		**Relaxed**		
		
		**Precision**	**Recall**	**F1-score**

**Pipeline**	No	95.06	88.95	91.90
**Pipeline**	Yes	**99.91**	88.51	93.87
**BioBERT**	No	99.68 ± 0.09	**92.58** ± 0.56	**95.99** ± 0.26
**SciBERT**	No	99.61 ± 0.15	91.77 ± 0.31	95.53 ± 0.10
**LLaMa-3.1-8B**	No	64.72	37.29	47.31
**gpt-oss-20b**	No	94.09	86.80	90.30
**BERN2**	Yes	42.91	25.44	31.95

Each metric (precision, recall, F1-score) expressed as percentage. Strict = indicates the complete span must match to be counted as true positive (TP), relaxed = at least one character must match between spans to be counted as TP. Evaluation done excluding all entities that define the (super)kingdoms of bacteria, archaea and fungi as these are the most common found entities and including these would inflate numbers.

Our two deep learning models were trained on labels generated by the pipeline with normalisation applied to the training and validation datasets (silver-standard). Our fine-tuned BioBERT and SciBERT models achieve strict F1-scores of 94.42 and 94.43, respectively, with relaxed F1-scores of 95.99 (BioBERT) and 95.53 (SciBERT). This compares with the LLaMa-3.1-8B and gpt-oss-20b LLMs achieving relaxed F1-scores of 47.31 and 90.30%, respectively, using few-shot prompting. The gain in performance over other models comes from an increased recall of the BERT-based deep learning models, with precision closely matched to the pipeline with normalisation (99.91% vs 99.61–99.68%). The variance of the SciBERT model (across 9 models) is lower than that of the BioBERT model (Table 3), indicating it is more stable across different randomisations.

We evaluate the performance of the BERN2 model on the test set and compare it with our microbiome-specific models. As not all species are microbial entities, and hence there can be many FPs, we evaluate BERN2 using the recall only. BERN2 achieves a relaxed recall of 25.44%, which is below our dictionary-based annotation pipeline (88.51–88.95%) and also not reaching the same level as our dedicated microbiome NER models (recall 91.77–92.58%).

### 3.3 Model evaluation for entity normalisation

Each of the annotations in the gold-standard test set (*n = *288 documents) was also given standard identifiers by two independent human researchers. The inter-annotator agreement across the 2041 unique annotations was 88.31% (F1-score) for EL. Disagreements were settled by a third arbiter as for NER to obtain a final test set with identifiers for each annotation. The pipeline achieved an accuracy for EL of 89.64% across all 20,486 annotations, dropping to 69.28% when considering each unique annotation only once (see Table 4). 5 deep learning models were evaluated for entity normalisation, 3 separate pre-trained models and our 2 fine-tuned models. These models have an overall accuracy for the top predicted identifier of 96.70–97.36% (Acc@1), increasing to 98.54–98.78% when a correct match is found in the top 5 predicted identifiers (Acc@5). For the unique accuracy, our fine-tuned models have an Acc@1 of 90.84–90.98% with the pre-trained models not far behind at 90.05–90.74%. At Acc@5, the pre-trained BioBERT model (95.00%) performs slightly better than our fine-tuned SciBERT (94.86%) and fine-tuned BioBERT (94.76%), with all models capable of accuracies >94%. Across all evaluations our fine-tuned SciBERT is top for the Acc@1 and second (after pre-trained BioBERT) for Acc@5. While the fine-tuned models show considerable improvement over pre-trained ones for NER, for EL using BioSyn no improvement was seen.

**Table 4:** Performance metrics (accuracy of top identifier (Acc@1) and top 5 identifiers (Acc@5) for different models evaluated on the manually annotated test set (*n = *288 full text articles) for entity normalisation.

		All		Unique	
Model	Fine-tuned	Acc@1	Acc@5	Acc@1	Acc@5
**Pipeline**	N/A	89.64	N/A	69.28	N/A
**BioBERT**	No	97.13	**98.78**	90.74	**95.00**
**SciBERT**	No	96.91	98.54	90.25	94.66
**SapBERT**	No	96.70	98.68	90.05	94.02
**BioBERT**	Yes	96.79	98.63	90.84	94.76
**SciBERT**	Yes	**97.36**	98.70	**90.98**	94.86

Different model embeddings (existing BioBERT (Lee *et al.* 2020), SciBERT (Beltagy *et al.* 2019), and SapBERT (Liu *et al.* 2021) models and our fine-tuned models) were used to train BioSyn (Sung *et al.* 2020) in hybrid mode.

### 3.4 Using the NER and EL models for literature review

The 6927 full-text articles were annotated using the NER (fine-tuned BioBERT) and EL (fine-tuned SciBERT embedding of the entity) pipelines, with the top candidate selected from the list of possible identifiers for each annotation. This resulted in a total of 182 064 entities in these documents, of which 6014 were unique, and specifically 126 842 entities (4878 unique) were found in the results sections. Resulting in a median number of reported unique entities in results sections of 30 (interquartile range 14–49), with a range of 0–578. Of the articles with at least one annotation identified in the results section text, 90% of articles have between 4 and 109 unique annotations mentioned.

**Figure 1:**
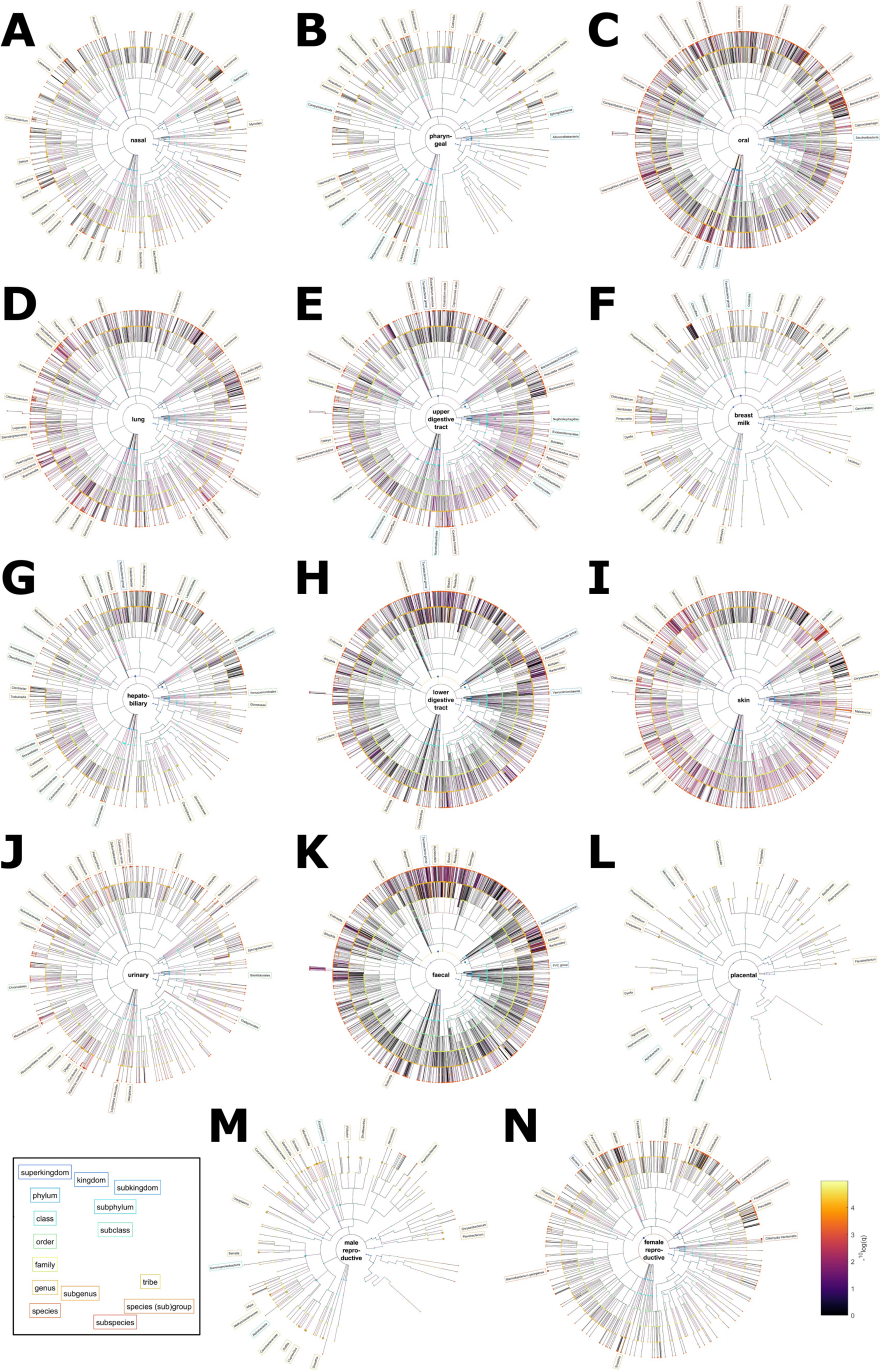
Taxonomic tree visualisation of domain-associated microbes. (A) Nasal. (B) Pharyngeal. (C) Oral. (D) Lung. (E) Upper gastrointestinal. (F) Breast milk. (G) Hepatobiliary. (H) Lower gastrointestinal. (I) Skin. (J) Urinary. (K) Faecal. (L) Placental. (M) Male reproductive. (N) Female reproductive microbiome. Individual high resolution figures for each domain can be found in the Supplementary Information, available as supplementary data at *Bioinformatics* online. Colour of the nodes relate to the taxonomic rank (see legend). The colour of the edges is proportional to the -log10 of the q-value (higher meaning more significant). The top microbial entities associated with each domain are visualised around each graph and coloured based on the taxonomic rank.

**Figure 2:**
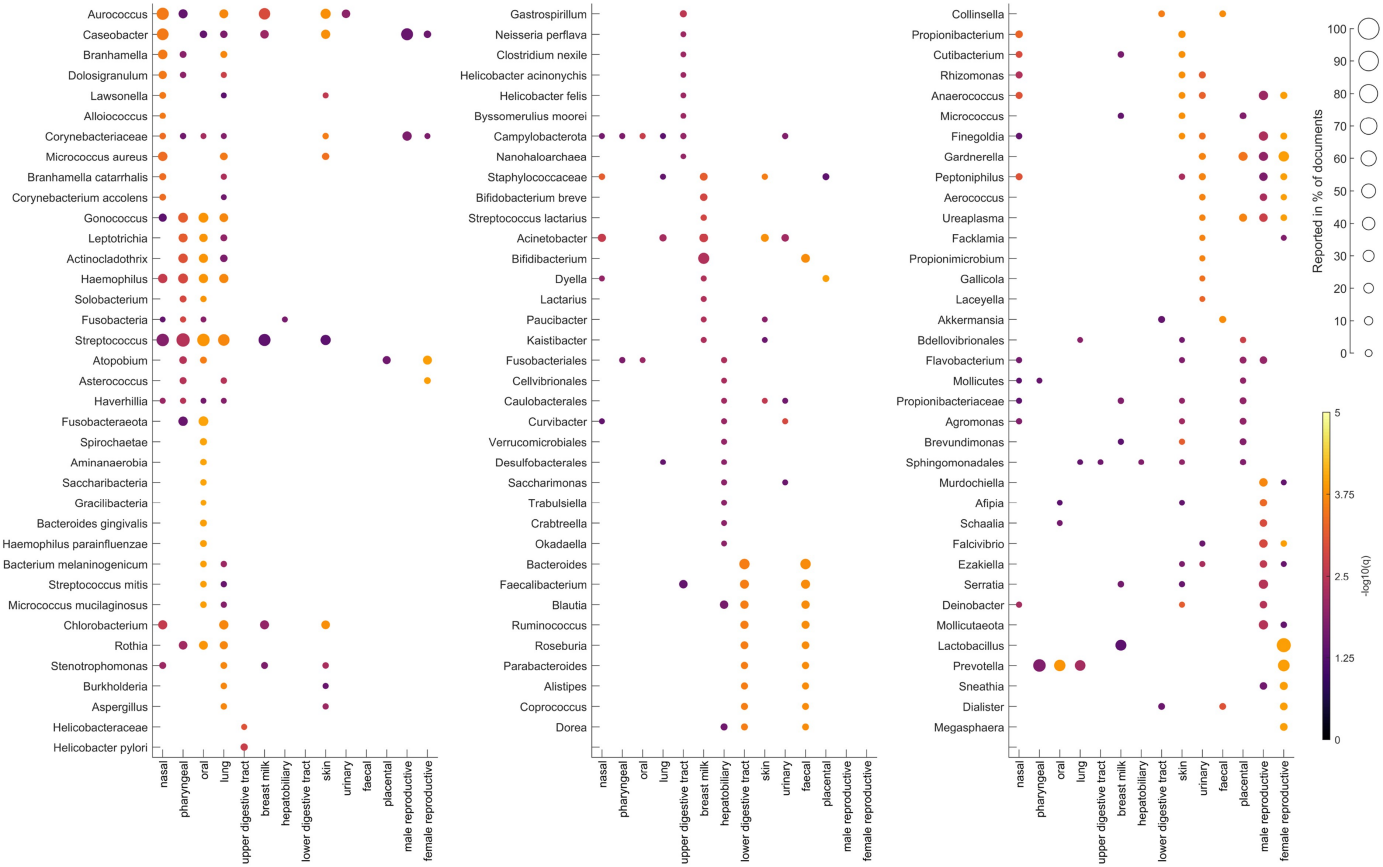
Heat map visualisation of the top 10 microbes for each of the 14 domains. Microbes with count >1 and *Q*-value ≤0.05 are visualised. The size of the marker indicates the proportion of documents within the domain that report the microbe. The marker colour indicates the −log10 *Q*-value (higher meaning more significant).

Figure 1 displays the taxonomic trees for each of the 14 domains. Across all trees, most of the significant taxa (*q* < 0.05) are either genera or species, followed by families and orders. The top 10 features per domain are given in Fig. 2, with any feature that is significant in any domain and present in any domain’s top 10 visualised. This illustrates the similarity of certain domains that can also be appreciated from the taxonomic trees, but here from the view of individual microbes with *Lactobacillus*, *Streptococcus*, and *Prevotella* the most commonly reported taxa amongst those that are significantly overrepresented in at least one domain.

#### 3.4.1 Common microbes

*Lactobacillus* was the most commonly reported taxon within a specific domain, with 73% of articles relating to the female reproductive (vagina, cervix, endometrium, but not urinary) microbiome reporting it in the results sections. It is significantly (*Q = *1.18×10^–4^) overrepresented in this domain compared to what would be expected at random. The only other domain in which it is significantly associated with is breast milk (human milk, colostrum, lactation) (*Q = *4.59×10^–2^), in 45% of articles. Within this genus *L. iners*, *L. crispatus*, *L. gasseri*, and *L. jensenii* (all at *Q = *1.57×10^–4^) were the most common species (26–46% of articles), with *L. johnsonii* (*Q = *2.08×10^–2^) and *L. psittaci* (*Q = *2.97×10^–4^) reported by 1–3% of studies on the female reproductive microbiome.

*Streptococcus* was found robustly associated (*Q = *1.43×10^–4^ to 4.98×10^–2^) with the oral (mouth, saliva), lung (lung, respiratory tract), pharyngeal, nasal (including nasopharynx), breast milk, and skin (skin, toe, foot, elbow fold, forehead) microbiomes (39–66% of articles). Individual *Streptococcal* species were reported in 0.3–7% of articles, with *S. mitis*, *S. salivarius*, *S. crista*, *S. vestibularis*, *S. peroris*, and *S. australis* reported by studies on the oral microbiome and at least one other domain (mostly lung, but also skin and upper digestive tract (oesophagus to duodenum)).

*Prevotella* was most significantly reported in the female reproductive microbiome literature (*Q = *1.18×10^–4^, 51.6%), but also with oral, lung, and pharyngeal studies (*Q = *1.43×10^–4^ to 1.70×10^–2^, 42–59%). However, the species within the genus showed no overlap between these domains, with *P. bivia*, *P. amnii*, *P. timonensis*, *P. buccalis*, *P. disiens*, and *P. bergensis* (*Q = *1.57×10^–4^ to 5.66×10^–3^) reported in studies of the female reproductive microbiome, and *P. melaninogenica*, *P. pallens*, *P. oris*, *P. salivae*, and *P. jejuni* (*Q = *1.19×10^–4^ to 4.77×10^–2^) in common between oral and lung microbiomes (with no species significantly reported more often for the pharyngeal microbiome).

Several other genera (e.g. *Neisseria*, *Haemophilus*, *Streptobacillus*) are in common between the nasal, pharyngeal, oral, and lung microbiomes, reflecting that these microbiomes are closely connected. Though there is some overlap with other domains for other taxa such as *Corynebacteriaceae* (skin, and male (testicle, semen, but not urinary) and female reproductive microbiomes) and *Campylobacterota* (upper digestive tract, urinary), with these taxa likely representing different lower rank taxa between these different domains. Overall, the most closely overlapped domains are faecal (faecal, stool) and lower digestive tract (jejunum to anus) microbiomes that have 462 different taxa associated significantly with at least one of these, of which 159 taxa (34%) are significantly associated with both. Despite that it appears the nasal, pharyngeal, oral, and lung microbiomes have a lot of overlap (Fig. 2), in practice the ratio between overlapped taxa over the total is in the order of 9–13% (with oral and nasal having 4% overlap). Similar to that of the female and male reproductive systems (18/151, 12%), and the male reproductive (not including urinary) and urinary microbiomes (11%). The reproductive, urinary, and placental microbiomes have *Gardnerella* and *Ureaplasma* in common, with the male and female reproductive system and urinary microbiomes sharing other common taxa with skin including *Anaerococcus*, *Finegoldia*, and *Peptoniphilus*.

#### 3.4.2 Domain specific microbes

Other taxa are only found overrepresented in a single domain. For example, a number of taxa are significant only for the oral microbiome such as the phyla *Spirochaetota*, *Synergistota*, *Saccharimonadota*, and *Altimarinota* (all *Q = *1.08×10^–4^). These were recognised in the text with different synonyms, e.g. for *Spirochaetota* (txid : 203,691) only one article used this nomenclature. The majority used *Spirochaetes* (*n = *132), with *Spirochaetota* (*n = *15), *Spirochaetae* (*n = *11), and *Spirochaeota* (*n = *2) used more often than the current accepted name.

The upper gastrointestinal tract also had considerable taxa that were found to be significantly associated only with this domain including *Helicobacteriaea*, *Helicobacter*, *H. pylori* (with 4 articles using synonym *Camphylobacter pylori*), *H. acinonychis*, *H. felis*, and *H. hominis* (*Q = *9.68×10^–4^ to 1.22×10^–2^), as well as *Neisseria perflava*, *Tyzzerella nexilis*, *Dictyonema moorei*, and *Nanohaloarchaea*.

Several *Bifidobacteria* were significantly reported more in the breast milk microbiome, such as *B. breve*, *B. choerinum*, *B. biavatii*, and *B. saguini* (*Q = *1.46×10^–3^ to 4.16×10^–2^). Also, significantly higher in only breast milk were *Streptococcus lactarius*, the fungus *Lactarius*, and *Plesiomonas*.

Finally, the hepatobiliary (liver, pancreas, bile duct, gall bladder) microbiome contains several significantly associated taxa at the order, *Cellvibrionales*, *Verrucomicrobiales*, and *Anaeroplasmatales* (*Q = *5.12×10^–3^ to 1.64×10^–2^), and genus levels, *Trabulsiella*, *Crabtreella*, *Okadaella*, and *Pseudochrobactrum* (*Q = *1.23×10^–2^ to 1.58×10^–2^). A complete table of all significantly associated microbes (common and unique) can be found in the Supplementary Materials, available as supplementary data at *Bioinformatics* online.

## 4 Discussion

This work is the first to create microbiome-specific corpora and algorithms for text mining research. Upon peer review, we will share the annotated Open Access documents that have licenses permitting redistribution in two ways. First, we share the training set with labels from our pipeline freely. Second, our gold-standard test set is split into a private (50 documents) and public (remaining documents) allocation. The private set is hosted on CodaLab with unannotated and normalised versions of the documents available for others, this is set up to allow benchmarking against these documents and to prevent data leakage of test data into models. The public set can be used to locally evaluate models, however with the risk that these data can be ingested into the training of LLMs (data leakage). Like the training set, we also share all documents with annotations and linked identifiers that made up our dataset for literature review, these documents were annotated by our DL algorithms (and not the pipeline). Our corpora are larger than most bioNLP corpora (Rosário-Ferreira *et al.* 2021), and compared with species-only corpora (Gerner *et al.* 2010, Pafilis *et al.* 2013) our training corpus contains 20× more annotations. The number of full-text documents contained in our corpora also exceeds that of the full-text (Gerner *et al.* 2010) and abstract-only (Pafilis *et al.* 2013) species corpora. The results sections alone from the literature review corpus contain 182 064 annotations (6014 unique), thus providing a valuable resource of additional training data for training NER and EL algorithms for recognising microbial entities.

The training corpus was annotated using the pipeline and used as input to train DL algorithms as an alternative to dictionary searching combined with rule-based annotation. The pipeline algorithm itself has been used separately as a first-pass approach to annotate microbiota followed by human review for a meta-analysis of microbiome research on malnutrition in African populations (Mweetwa *et al.* 2026). We have previously demonstrated how ‘silver’ annotated training data can achieve accurate NER algorithms for domains that have no training data available such as metabolites (Yeung *et al.* 2022) and enzymes (Wang *et al.* 2024). Here we showed a performance gain of over 2% for NER of the DL algorithms (BioBERT achieving an F1-score of 0.96), and over 30% for EL (SciBERT achieving an accuracy of 91%). While the DL models have slightly lower precision than the pipeline (-0.3%) for NER, this is negligible compared with the gain in recall and speed. This indicates that the DL models learn how to identify entities in the sentence rather than only identifying entities present in the training set. The pipeline takes on average 100 seconds compared with the BERT-based DL models taking only 7 seconds per document, while both methods were considerably faster than LLMs (674–1822 seconds per document, see Supplementary Information, available as supplementary data at *Bioinformatics* online). A challenging aspect of our test set is that 7.73% of annotations in the test set can only be found here and are not contained in the training set. The higher recall of the DL models demonstrated that these models are able to recognise additional entities not found using exact matching, including some spelling errors in names that were correctly resolved to the right taxonomic identifier with the EL models. This model can also be used in conjunction with others (mixture of experts) for annotation of multiple categories of entities. For example, one of the limitations of our prior work on metabolite NER Yeung *et al.* 2022 demonstrated that a small proportion of microbiota are falsely recognised as metabolites. This likely occurs due to the similarity of certain tokens such as for example ‘butyr’ in the metabolite butyrate and the microbial genus *Butyrococcus*.

Filtering the data for any significant taxa (*Q* < 0.05) reported at least by 2 independent studies showed that a coverage of at least 50% of all these significant taxa can be obtained with only 5 studies for most domains. The exceptions were faecal (with 5 studies combined mentioning 46% of the 300 significant taxa), skin (38%, 298 significant taxa), and upper digestive tract (38%, 114 significant taxa), with 6, 9 and 8 articles needed to have a coverage of at least 50% for each of these respectively. While this is logically related to the number of unique taxa reported per domain, others with similar numbers (e.g. oral with 292 significant taxa) can have a coverage of over 50% with fewer articles. Supplementary Information, available as supplementary data at *Bioinformatics* online contains an extended discussion on how this can be used to assist the literature review stage of a new study.

### 4.1 Limitations

One limitation of our methods are that they were not trained to recognise viruses, but only trained on archaea, bacteria, and fungi. This was recognised by Mweetwa *et al.* 2026 whom annotated the viruses manually. However, as viral genomes are typically much smaller and lacking a universal marker gene (e.g. 16S or 18S rRNA, ITS) they are not commonly captured by the bioinformatic pipelines used to compare sequences with libraries. With the released corpora and pipeline, viruses can be added to the pipeline for these documents to be re-annotated. We share our code and models (following peer review) to retrain or fine-tune the DL models thus these can be updated to include viruses. However, since our test set does not contain viruses, a new test set would need to be created to evaluate the accuracy of the updated models.

We demonstrated that BERT-based architectures outperform open-weight, locally-deployable LLMs for microbiome NER. Related work has shown that LLMs fail to generalise to out-of-domain tasks such as entity recognition, with no LLM coming close to BERT-based models for NER (Chen *et al.* 2023, Hu *et al.* 2024, Jahan *et al.* 2024) or EL (Jahan *et al.* 2024). The BERT-based models are capable of annotating entire documents in seconds, however this does require such models to be loaded into memory beforehand. Hence, annotating a single document will take considerably longer than annotating many documents in one go, since the model loading time cannot be amortised across multiple inputs. Likewise, loading the EL model also costs time but inference is fast (as shown in the Supplementary Information, available as supplementary data at *Bioinformatics* online).

Utilising these models for literature summarisation has several aspects that have room for improvement. First, it is dependent on defining a set of articles relevant to a new study, this human task would ideally be done in a systematic manner to determine appropriate inclusion and exclusion terms. The search queries used here can be adapted by adding additional filters such as e.g. filtering faecal microbiome studies for specific diseases of interest, though the output of our models remain dependent on what is reported by the authors in each article (hence depends on their reported findings). Our models here focus on first recognising the entities and second assigning identifiers to them. They do not perform relation extraction, thus do not provide any context of how the identified microbe is related to the study outcome (e.g. is it higher/lower compared to controls). In other contexts, LLMs have shown capabilities to reliably extract relations (Chen *et al.* 2023, Choi *et al.* 2026, Jahan *et al.* 2024) thus these could be explored should users want to automate this step.

## 5 Conclusion

In summary, this work contributed a novel dictionary- and rule-based automatic pipeline for microbiome NER and EL with almost perfect precision. The best performing DL model was BioBERT with a state-of-the-art performance (F1-score >0.95) for microbiome NER that considerably speeds up the annotation process. We have shown the application of these models for assisting with literature review and capability to meta-analyse the existing literature to determine taxa that are significantly over-reported in certain domains.

## Supplementary Material

btag418_Supplementary_Data

## Data Availability

All codes are available for automatic annotation and model training, with instructions on how to deploy the model on new text, from GitHub at https://github.com/omicsNLP/microbELP and Zenodo at https://dx.doi.org/10.5281/zenodo.20613467. The redistributable, annotated training set and unannotated test set are made available from Zenodo at https://x.doi.org/10.5281/zenodo.17305410 with the redistributable, human-labelled test set hosted as benchmark on Codabench at https://www.codabench.org/competitions/10913/ (for NER only) and at https://www.codabench.org/competitions/11581/ (for NER+EL) for evaluation. The annotated documents for all available literature are hosted separately on Zenodo at https://dx.doi.org/10.5281/zenodo.17288826.
