# Supplementary material for: Microbial named entity recognition and normalisation for AI-assisted literature review and meta-analysis
Source: Bioinformatics. 2026 Jun 20;42(7):btag418. doi: 10.1093/bioinformatics/btag418 (PMC13342716; doi:10.1093/bioinformatics/btag418)
Supplement: btag418_Supplementary_Data [file btag418_supplementary_data.pdf]

Supplementary Materials

Microbial Named Entity Recognition and Normalisation for AI-assisted Literature Review and Meta-Analysis

Dhylan Patel,[1,2,a] Antoine D. Lain,[1,a] Avish Vijayaraghavan,[1,3,a] Nazanin Faghih Mirzaei,[1,a] Monica N. Mweetwa,[1,4] Meiqi Wang,[1] Tim Beck[5,6] and Joram M. Posma[1,6,] *

[1]Section of Bioinformatics, Division of Systems Medicine, Department of Metabolism, Digestion and Reproduction, Imperial College London, W12 0NN, United Kingdom, [2]Department of Life Sciences, Imperial College London, SW7 2AZ, United Kingdom, [3]UKRI Centre for Doctoral Training in AI for Healthcare, Department of Computing, Imperial College London, SW7 2AZ, United Kingdom, [4]Tropical Gastroenterology and Nutrition Group (TROPGAN), School of Medicine, University of Lusaka, Zambia, [5]Centre for Health Informatics, School of Medicine, University of Nottingham, NG7 2RD, United Kingdom, [6]Health Data Research (HDR) UK, NW1 2BE, United Kingdom and [a]These authors contributed equally.

*Corresponding author. jmp111 [at] ic.ac.uk

Abstract

Contents:
Supplementary Methods:

- Query for obtaining the training corpus
- Processing of the NCBI taxonomy
- Rule-based annotation pipeline
- Calculation of null model and P-value calculation for domain-specific nodes

Extended results for time taken per model (averaged per document). Extended discussion on the use of the tool for literature review. Larger figures of the individual panels of the main figure containing all taxonomic trees for the different domains:

- nasal (including nasopharynx) microbiota (Figure S2)
- pharyngeal microbiota (Figure S3)
- oral (mouth, saliva) microbiota (Figure S4)
- lung (including respiratory tract) microbiota (Figure S5)
- upper gastrointestinal (oesophagus to duodenum) microbiota (Figure S6)
- breast milk (human milk, colostrum, lactation) microbiota (Figure S7)
- hepatobiliary (liver, pancreas, bile duct, gall bladder) microbiota (Figure S8)
- lower gastrointestinal (jejunum to anus) microbiota (Figure S9)
- skin (skin, toe, foot, elbow fold, forehead) microbiota (Figure S10)
- urinary microbiota (Figure S11)
- faecal (faecal, stool) microbiota (Figure S12)
- placental microbiota (Figure S13)
- male reproductive (testicle, semen, but not urinary) microbiota (Figure S14)
- female reproductive (vagina, cervix, endometrium, but not urinary) microbiota (Figure S15)

© The Author . Published by Oxford University Press. All rights reserved. For permissions, please e-mail: journals.permissions@oup.com

1. Supplementary Methods

1.1. Data - training corpus

The following search query was used for obtaining all 'faecal microbiome' articles: *(metagenomics[Abstract] OR metagenomic[Abstract] OR metagenome[Abstract] OR microbiome[Abstract] OR microbiomic[Abstract] OR 16S[Abstract] OR meta-genome[Abstract] OR microbial[Abstract] OR microbes[Abstract] OR metagenomics[Title] OR metagenomic[Title] OR metagenome[Title] OR microbiome[Title] OR microbiomic[Title] OR 16S[Title] OR meta-genome[Title] OR microbial[Title] OR microbes[Title]) AND (faecal[Abstract] OR faeces[Abstract] OR stool[Abstract] OR feces[Abstract] OR fecal[Abstract] OR faecal[Title] OR faeces[Title] OR stool[Title] OR feces[Title] OR fecal[Title]) AND (16S[Abstract] OR rRNA[Abstract] OR sequencing[Abstract] OR shotgun[Abstract] OR Illumina[Abstract] OR MiSeq[Abstract] OR PCR[Abstract] OR 16S[Title] OR rRNA[Title] OR sequencing[Title] OR shotgun[Title] OR Illumina[Title] OR MiSeq[Title] OR PCR[Title]) AND (abundance[Abstract] OR abundant[Abstract] OR diversity[Abstract] OR abundance[Title] OR abundant[Title] OR diversity[Title]) AND (human[Abstract] OR patients[Abstract] OR subjects[Abstract] OR participants[Abstract] OR human[Title] OR patients[Title] OR subjects[Title] OR participants[Title]) NOT (mouse[Abstract] OR mice[Abstract] OR rat[Abstract] OR rats[Abstract] OR dog[Abstract] OR dogs[Abstract] OR animal[Abstract] OR cell culture[Abstract] OR dose[Abstract] OR review[Title] OR proteomics[Abstract] OR diet[Abstract] OR proteomic[Abstract] OR proteome[Abstract] OR transcriptomics[Abstract] OR transcriptomic[Abstract] OR transcriptome[Abstract] OR mouse[Title] OR mice[Title] OR rat[Title] OR rats[Title] OR dog[Title] OR dogs[Title] OR animal[Title] OR cell culture[Title] OR dose[Title] OR diet[Title] OR proteomics[Title] OR proteomic[Title] OR proteome[Title] OR transcriptomics[Title] OR transcriptomic[Title] OR transcriptome[Title]) AND ("01/01/2000"[Publication Date] : "3000"[Publication Date]).* Where the grey colour in the query was replaced for each of the domains with synonyms for each of the domains.

1.2. Data - NCBI taxonomy

Several data cleaning steps were performed to the NCBI taxonomy data: main taxonomic names were extracted from the name fields with species as lowest level. Data with lower taxonomic ranks than species were pruned to their species names, with subspecies given the name of the [genus subspecies] pair as 'species' name, with species names extracted from the full names associated with the forma, species group and species subgroup ranks. Data without a rank ('no rank') was assigned a taxonomic rank based on the (grand)parent and (grand)child nodes with a likely rank inferred from this. This means an entry with 'no rank' whose parent is a genus and with (grand)children from taxonomy ranks such as 'isolate', 'strain', etc. were assigned to 'species'.

Duplicate names within the same identifier were removed, in addition to the following rules to remove entries: any 'species' without a space in the name, 'species' where the cleaned species name includes a '.' but not 'sp.', any uncommon ending (e.g. '-ic' or '-ing'), any entry where the name includes any of the following terms (group, subdivision, symbiont, cluster, biofilm, candidate, candidatus, unclassified, environmental, witches, marine, enrichment), any entry with a country name or country adjective, any entry with less than 4 lowercase letters, any entry where the cleaned name includes a colour, and any entry where the cleaned name includes the source (e.g. soil, sea(water), landfill) or a non-microbial species (e.g. penguin).

1.3. Rule-based annotation pipeline

The (full)text is scanned word for word for any exact matches to words (i.e. the entry *Escherichia coli* is split into two words) in the dictionary. If no match is triggered, the word is converted to Latin singular/plural forms (i.e. *Actinobacterial* to *Actinobacteria*) and checked again. If a match is triggered the words before and after are evaluated. If the word before is already annotated (e.g. a genus) and no punctuation can be found between both words they are combined as a single entity (case for species) to be matched against the names in the dictionary. If the word after triggers a match (without punctuation in between) they are combined and matched to entries in the dictionary. If the word before is a capital letter only, a period was removed after it, and the trigger word is lowercase it is assumed to be an abbreviated entity (e.g. *E. coli*). For abbreviated entities the prior annotations in the document are searched for any match for the full name, if there is a match the abbreviation inherits the ID from the full term, if there is no match then all matches in the dictionary are added (e.g. *M. epidermidis* mapping to *Micrococcus epidermidis* (txid1282) and *Macrococcus epidermidis* (txid1902580)). Once the document is fully annotated it iterates over all annotations with more than one assigned identifier of different taxonomic ranks. For those entries (e.g. *Actinobacteria* phylum and class) the other annotations in the sentence are checked, if only one of the ranks is represented in the other annotations this is the only ID selected for the annotation with more than one. If both or neither are matched, all IDs remain assigned to the entity. The full workflow of corpus creation is given in Figure S1.

1.4. Taxonomic tree

A combined set of all taxonomic identifiers found in any section of any document across the 14 domains was compiled and used to create a 'reference' taxonomic tree. The taxonomic identifiers were matched against the NCBI Taxonomy database (see 'Data – NCBI Taxonomy' section) and their parent IDs extracted. Any parent ID not yet in the list is then searched in the same database, until either it is part of the list or the parent ID's rank is (super)kingdom (highest rank we include). Each entry in the reference taxonomic tree then gets the number of documents (count) it was found in as node weight.

For the domain specific trees, the structure of the reference tree is used with 0 counts for all nodes. Then the individual counts of microbes across domain-specific articles are added as node weights. Secondly, we traverse the tree, starting at the lowest taxonomic rank and add weighted counts to the parent node. The weighting takes place based on the number of child nodes the parent has in the complete taxonomy database, not only in the reference tree. As example, a genus with 10 children in the database and 3 children in

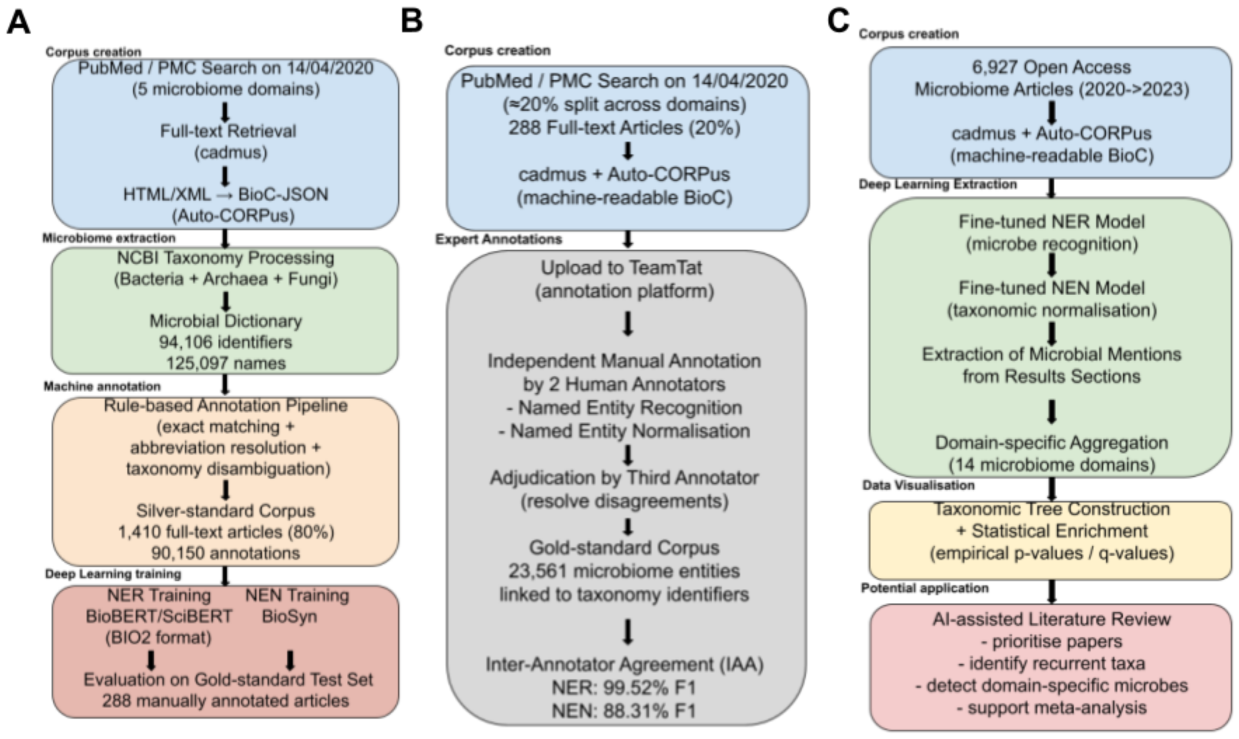

Fig. S1: Overview of the microbiome-specific corpus creation, gold-standard annotation, and AI-assisted literature review workflow. (A) Full-text microbiome articles were retrieved from PubMed Central and converted into machine-readable BioC format using cadmus and Auto-CORPus. A curated microbial dictionary derived from the NCBI Taxonomy database was used in a rule-based annotation pipeline to generate a silver-standard corpus for training deep learning models for named entity recognition (NER) and named entity normalisation (NEN). (B) A gold-standard test corpus was created from 288 full-text microbiome articles. Articles were retrieved using cadmus, converted into BioC XML format with Auto-CORPus, and uploaded into TeamTat for annotation. Two independent annotators manually annotated microbial entities and linked taxonomic identifiers, with disagreements resolved by a third annotator. The resulting corpus achieved inter-annotator agreement scores of 99.52% for NER and 88.31% for NEN. (C) The fine-tuned NER and NEN models were applied to another 6,927 Open Access microbiome articles to extract and normalise microbial mentions from results sections. Aggregated taxa were used to construct taxonomic trees and identify domain-specific microbial associations, enabling AI-assisted literature review and meta-analysis.

the 'reference' will get an updated weight of their original node weight (counts) plus the counts of the 3 children divided by 10 children overall. If the genus has a weight of 5, and the 3 children have weights of 1, 0 and 3, respectively, the updated score of the genus is $5 + \frac{1}{10} + \frac{0}{10} + \frac{3}{10} = 5.4$.

Once the taxonomic tree is completely traversed and node weights updated, we then determine an empirical estimate of the null distribution of the node weights by resampling (nr=99,999 times, random state set to 2 before starting) using the reference tree weights (per taxonomic rank) as sampling probability for each non-zero node in the domain tree. I.e. for all nodes of rank 'species' in the domain tree we first sum the counts ('sc') and find the identifiers of these nodes ('ni'). We then extract from the reference tree the counts for nodes with IDs contained in 'ni' as a vector, and normalise the vector ('vp') to a sum of 1 (probability). Random sampling with weights 'vp' is done 'sc' times for the rank, and these counts are added to the 'i^{th}' resampled tree. This same process is done for all ranks. Any rank between any of the main ranks ((super)kingdom, phylum, class, order, family, genus, species) is sampled together with the higher rank (e.g. subgenus with genus). Then the counts in the resampled tree 'i' is aggregated as described above, and across all 'nr' resampled trees we count for each node the number of times ('nt') the aggregated count of the node is larger or equal to the domain tree's aggregated count. This procedure allows us to estimate the proportion of times random sampling would result in higher counts than the actual value of the domain tree, and thus to calculate an empirical p-value for each node in the graph. The empirical P-value is defined as $\frac{nt}{nr} \forall nt > 0$ and $\frac{1}{nr+1} \forall nt = 0$. All P-values within each taxonomic rank are then adjusted for multiple testing using the Storey-Tibshirani False Discovery Rate (FDR, Q-value) given that they are independent at each rank but there is dependence between ranks. The Q-values are visualised on a $-log10$ scale, with significance indicated by Q<0.05 (or 1.30 on the $-log10$ scale). For any node in the graph, a significant Q-value indicates that this microbe is mentioned more than would be expected by random chance.

2. Supplementary Results

Table S1. Runtime assessment of all models reported in the main manuscript for both NER and NEN. BioBERT models refer to our fine-tuned models, and LLMs refer to few-shot prompting.

Model	Resource	Total time (288 documents)	Time per document (s)
Pipeline (NER + NEN)	CPU	7h 58.1m	99.6
BioBERT (NER + NEN)	CPU	1h 48.5m	22.6
BioBERT (NER + NEN)	GPU	33.3m	6.9
BERN2 (NER + NEN)	CPU	2h 36.2m	32.5
BERN2 (NER + NEN)	GPU	48.5m	10.1
Pipeline (NER only)	CPU	7h 20.8m	91.8
BioBERT (NER only)	CPU	10.1m	2.1
BioBERT (NER only)	GPU	47.6s	0.2
gpt-oss-20b (NER only)	GPU	6d 1h 47.2m	1,822.3
LLaMa-3.1-8B (NER only)	GPU	2d 5h 55.9m	674.2
BioSyn on BioBERT (NEN only)	CPU	1h 44.5m	21.8
BioSyn on BioBERT (NEN only)	GPU	33.2m	6.9

3. Supplementary Discussion

The filtering of the taxonomic tree data to its most significant elements (nodes at which there is a significantly higher than expected count) can also be used to order relevant articles for the literature review stage of a new study. The way we envision this process can take two forms. The first is analogous to how it was done here, by obtaining machine-readable formats for all relevant articles through a PubMed search and obtaining XML versions of these full-text documents [Campbell et al., 2026], conversion of such documents to the machine-readable BioC-standard [Beck et al., 2022], followed by annotation using the NER and EL models contributed here. From these the reported taxa from results sections can be extracted and summarised, and a document ranking achieved based on the unique number of taxa each article contributes (the background set of microbes reported in any domain, or in specific domains can both be used for this purpose). For example, for the vaginal microbiome the combination of 3 studies [Andralojc et al., 2021, dos Anjos Borges et al., 2023, Kim et al., 2021] describes 51% of the microbes that we find significantly reported more often in this domain compared to others, this number which goes up to 83% with 10 articles (3.6% of the total of 281 articles in our corpus from this domain). This approach can lead to identifying targets to test in new data to reproduce earlier results, or to otherwise generate hypotheses.

The second approach starts the same to collect all relevant articles (with appropriate search terms to filter more relevant articles at the first stage), however rather than finding taxa that are reported more often than would be expected by random chance instead the results from a new study can be compared against the literature to find the relevant articles to inspect further. While there will not be a BioC-version of a manuscript in preparation, this is not needed for the algorithms to work. I.e. if the significant microbes are passed as text file to the EL it will output the taxonomic identifiers, and then based on the entire body of relevant literature a search can be conducted to find a set of articles that describe as much from the findings as possible. At the same time, this will also implicitly pinpoint any novel findings in a new study. For example, taking the most recent article in our corpus on the breast milk microbiome [Filatava et al., 2023], we can establish that different combinations of 4-5 articles from our corpus report the 22 unique taxa reported in Filatava et al., 2023 (published in December 2023), thus any of these combinations could form a logical first step for literature review. For example, of a set of 4 [Boix-Amorós et al., 2016, Lyons et al., 2022, Juárez-Castelán et al., 2022, Toubon et al., 2023] (April 2016 to May 2023), 2 of these were cited and of another [Juárez-Castelán et al., 2022] an article by the same authors was cited. Only one article published in the same year (May 2023) was not cited. Another combination that fully covers all reported microbes contains 5 articles [Boix-Amorós et al., 2016, Ojo-Okunola et al., 2019, Lopez Leyva et al., 2021, Lyons et al., 2022, Toubon et al., 2023], of which the same two were cited and Toubon et al., 2023 was not, but the other two were not cited however articles by the same authors were cited (likely covering similar cohorts). Using this approach to shortlist articles for review is dependent on adequately defining the relevant literature (human effort) with the steps after it automated. The automation can save researchers time with identifying which articles to read first by generating a list of priorities. With domains that have hundreds (only the breast milk, male reproductive, and placental microbiomes had under 100 articles) or thousands (the faecal microbiome portion of our corpus contains over 2,000 documents) of articles, this approach could save time for researchers when conducting their literature review, as noted in previous work [Mweetwa et al., 2025].

References

K. M. Andralojc, M. A. Molina, M. Qiu, B. Spruijtenburg, M. Rasing, B. Pater, M. A. Huynen, B. E. Dutilh, T. H. A. Ederveen, D. Elmelik, A. G. Siebers, D. Loopik, R. L. M. Bekkers, W. P. J. Leenders, and W. J. G. Melchers. Novel high-resolution targeted sequencing of the cervicovaginal microbiome. *BMC Biology*, 19(1), Dec. 2021. ISSN 1741-7007. doi: 10.1186/s12915-021-01204-z.

T. Beck, T. Shorter, Y. Hu, Z. Li, S. Sun, C. M. Popovici, N. A. R. McQuibban, F. Makraduli, C. S. Yeung, T. Rowlands, and J. M. Posma. Auto-CORPus: A Natural Language Processing Tool for Standardising and Reusing Biomedical Literature. *Frontiers in Digital Health*, 4(788124):788124, Jan. 2022. ISSN 2673-253X. doi: 10.3389/fdgth.2022.788124.

A. Boix-Amorós, M. C. Collado, and A. Mira. Relationship between milk microbiota, bacterial load, macronutrients, and human cells during lactation. *Frontiers in Microbiology*, 7, Apr. 2016. ISSN 1664-302X. doi: 10.3389/fmicb.2016.00492.

J. Campbell, A. D. Lain, and T. I. Simpson. cadmus: a robust pipeline for scalable retrieval of full-text biomedical literature. *bioRxiv*, 2026. doi: 10.64898/2026.05.16.725623. URL `https://www.biorxiv.org/content/early/2026/05/19/2026.05.16.725623`.

L. G. dos Anjos Borges, J. Pastuschek, Y. Heimann, K. Dawczynski, M. Bergner, R. Haase, J. Stubert, D. Olbertz, I. Plumeier, S. Kahl, A. K. Heroven, E. Schleußner, D. H. Pieper, and J. Zöllkau. Vaginal and neonatal microbiota in pregnant women with preterm premature rupture of membranes and consecutive early onset neonatal sepsis. *BMC Medicine*, 21(1), Mar. 2023. ISSN 1741-7015. doi: 10.1186/s12916-023-02805-x.

E. J. Filatava, Z. Liu, J. Xie, D.-B. Tran, K. Chen, N. El Habbal, G. Weinstock, Y. Zhou, and K. E. Gregory. The preterm human milk microbiota fluctuates by postpartum week and is characterized by gestational age and maternal bmi. *mBio*, 14(6), Dec. 2023. ISSN 2150-7511. doi: 10.1128/mbio.02106-23.

C. J. Juárez-Castelán, J. M. Vélez-Ixta, K. Corona-Cervantes, A. Piña-Escobedo, Y. Cruz-Narváez, A. Hinojosa-Velasco, M. E. Landero-Montes-de Oca, E. Davila-Gonzalez, E. González-del Olmo, F. Bastida-Gonzalez, P. B. Zárate-Segura, and J. García-Mena. The entero-mammary pathway and perinatal transmission of gut microbiota and sars-cov-2. *International Journal of Molecular Sciences*, 23(18):10306, Sept. 2022. ISSN 1422-0067. doi: 10.3390/ijms231810306.

S. Kim, H. Seo, M. A. Rahim, S. Lee, Y.-S. Kim, and H.-Y. Song. Changes in the microbiome of vaginal fluid after menopause in korean women. *Journal of Microbiology and Biotechnology*, 31(11):1490–1500, Nov. 2021. ISSN 1738-8872. doi: 10.4014/jmb.2106.06022.

L. Lopez Leyva, E. Gonzalez, C. Li, T. Ajeeb, N. W. Solomons, L. B. Agellon, M. E. Scott, and K. G. Koski. Human milk microbiota in an indigenous population is associated with maternal factors, stage of lactation, and breastfeeding practices. *Current Developments in Nutrition*, 5(4):nzab013, Apr. 2021. ISSN 2475-2991. doi: 10.1093/cdn/nzab013.

K. E. Lyons, C.-A. O. Shea, G. Grimaud, C. A. Ryan, E. Dempsey, A. L. Kelly, R. P. Ross, and C. Stanton. The human milk microbiome aligns with lactation stage and not birth mode. *Scientific Reports*, 12(1), Apr. 2022. ISSN 2045-2322. doi: 10.1038/s41598-022-09009-y.

M. N. Mweetwa, M. P. Kelly, and J. M. Posma. A meta-analysis of gut microbiome research in malnourished African populations: A natural language processing approach. *Philosophical Transactions of the Royal Society B*, in press, Dec. 2025. ISSN 1471-2970. doi: 10.1098/rstb.2025.0038.

A. Ojo-Okunola, S. Claassen-Weitz, K. S. Mwaikono, S. Gardner-Lubbe, D. J. Stein, H. J. Zar, M. P. Nicol, and E. du Toit. Influence of socio-economic and psychosocial profiles on the human breast milk bacteriome of south african women. *Nutrients*, 11(6):1390, June 2019. ISSN 2072-6643. doi: 10.3390/nu11061390.

G. Toubon, M.-J. Butel, J.-C. Rozé, I. Nicolis, J. Delannoy, C. Zaros, P.-Y. Ancel, J. Aires, and M.-A. Charles. Early life factors influencing children gut microbiota at 3.5 years from two french birth cohorts. *Microorganisms*, 11(6):1390, May 2023. ISSN 2076-2607. doi: 10.3390/microorganisms11061390.

4. Supplementary Figures

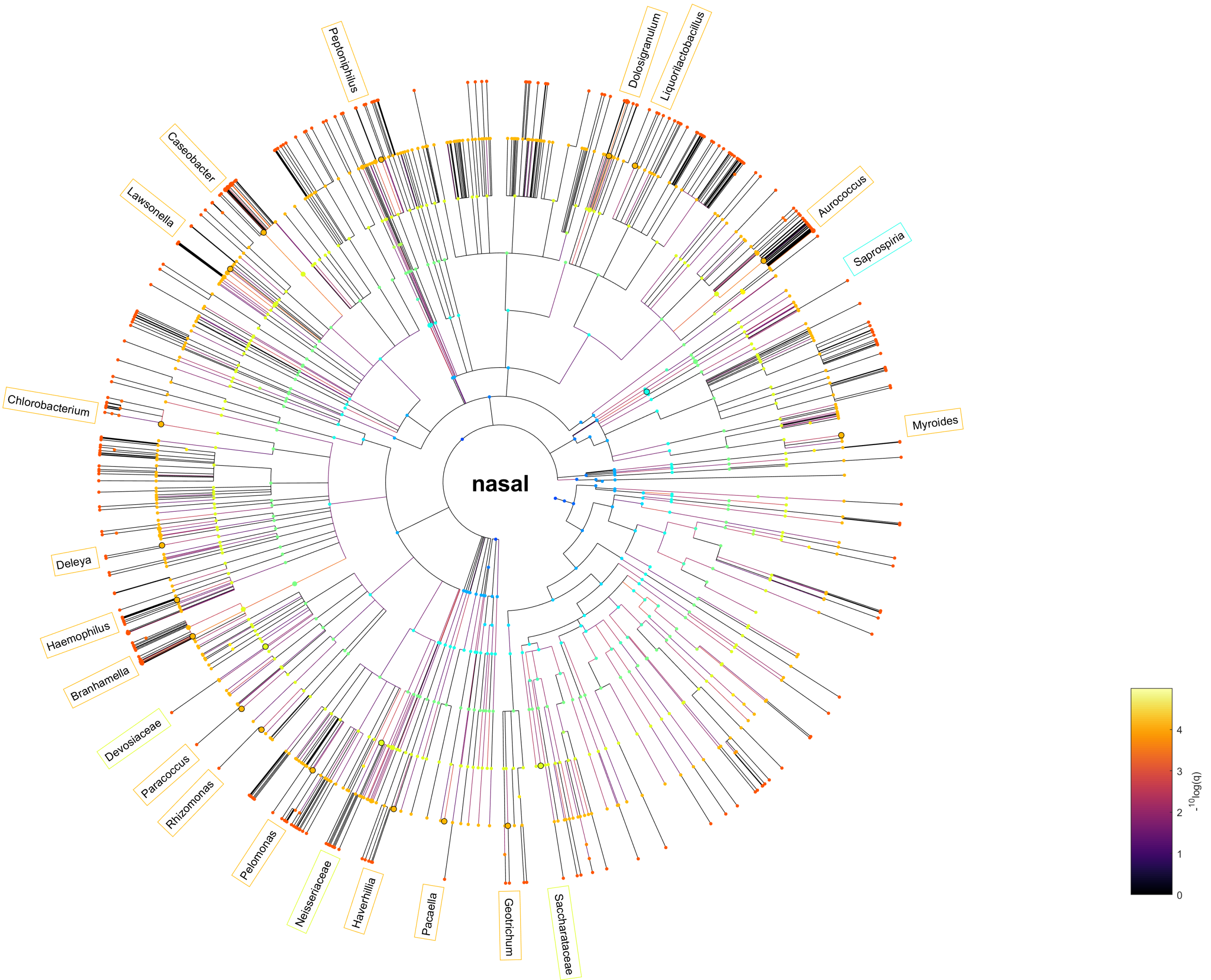

Fig. S2: Taxonomic tree visualisation of the nasal (including nasopharynx) microbiota. Colour of the nodes relate to the taxonomic rank. The colour of the edges is proportional to the -log10 of the q-value (higher meaning more significant). The top microbial entities associated with the domain are visualised around the graph and coloured based on the taxonomic rank.

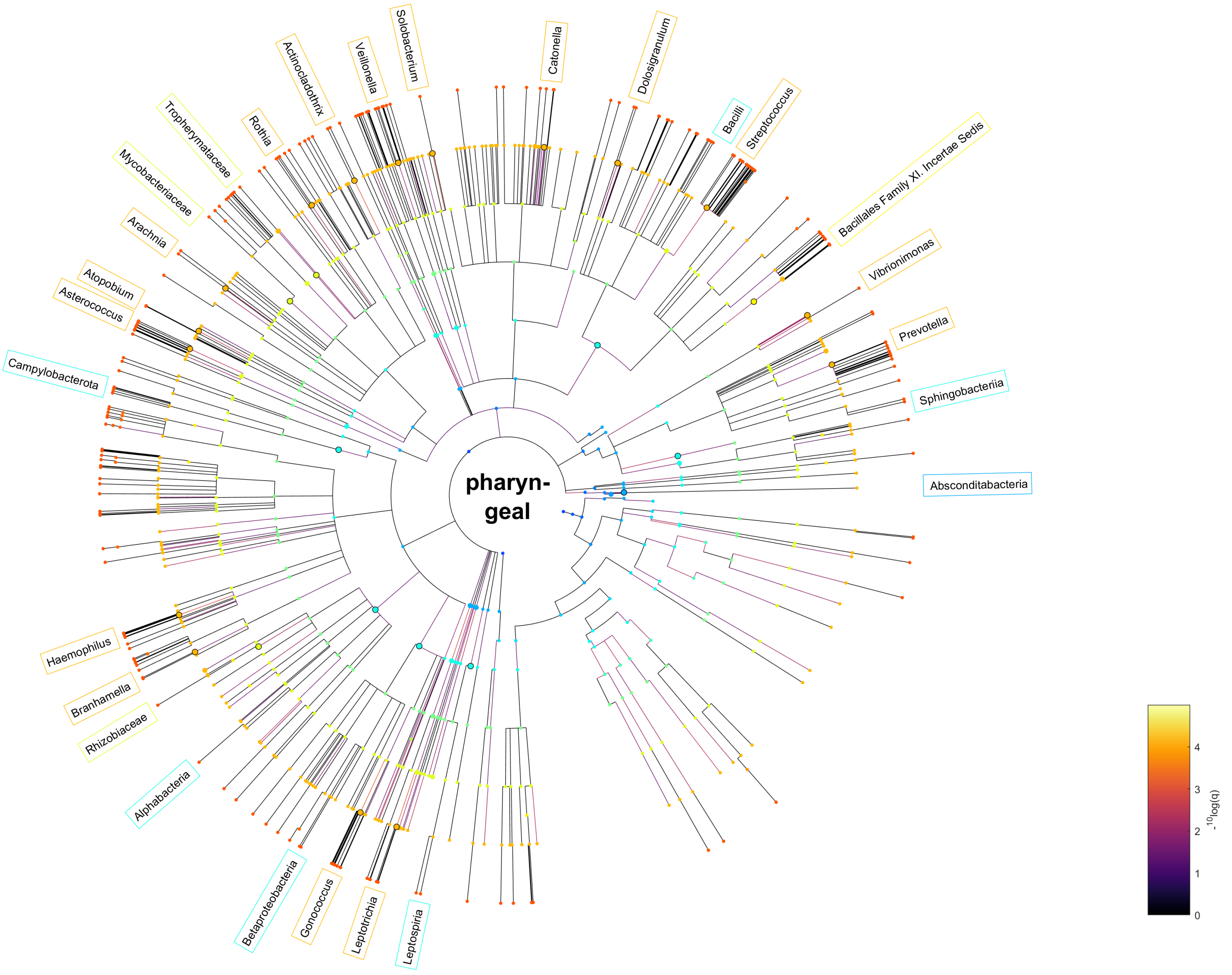

Fig. S3: Taxonomic tree visualisation of the pharyngeal microbiota. Colour of the nodes relate to the taxonomic rank. The colour of the edges is proportional to the -log10 of the q-value (higher meaning more significant). The top microbial entities associated with the domain are visualised around the graph and coloured based on the taxonomic rank.

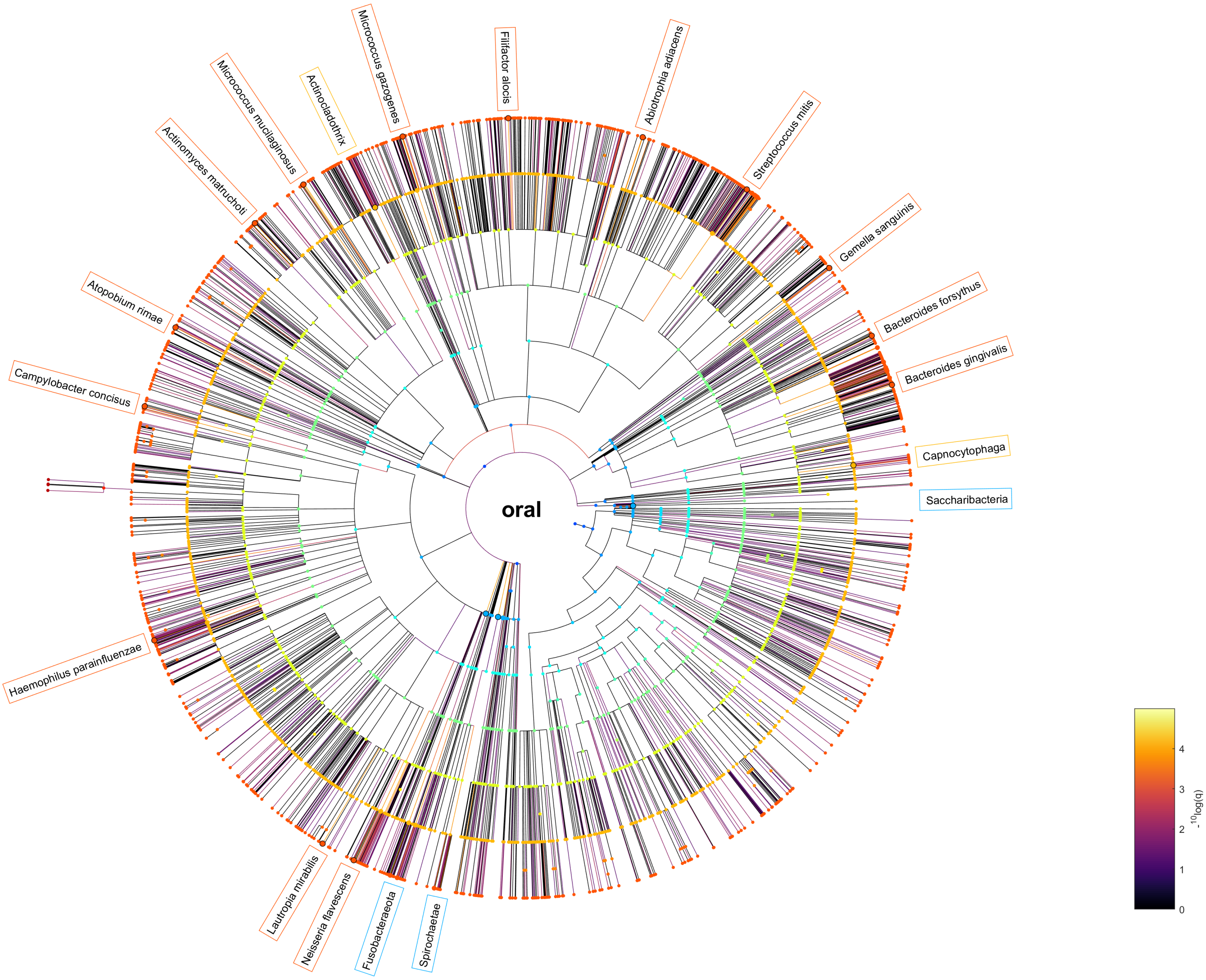

Fig. S4: Taxonomic tree visualisation of the oral (mouth, saliva) microbiota. Colour of the nodes relate to the taxonomic rank. The colour of the edges is proportional to the -log10 of the q-value (higher meaning more significant). The top microbial entities associated with the domain are visualised around the graph and coloured based on the taxonomic rank.

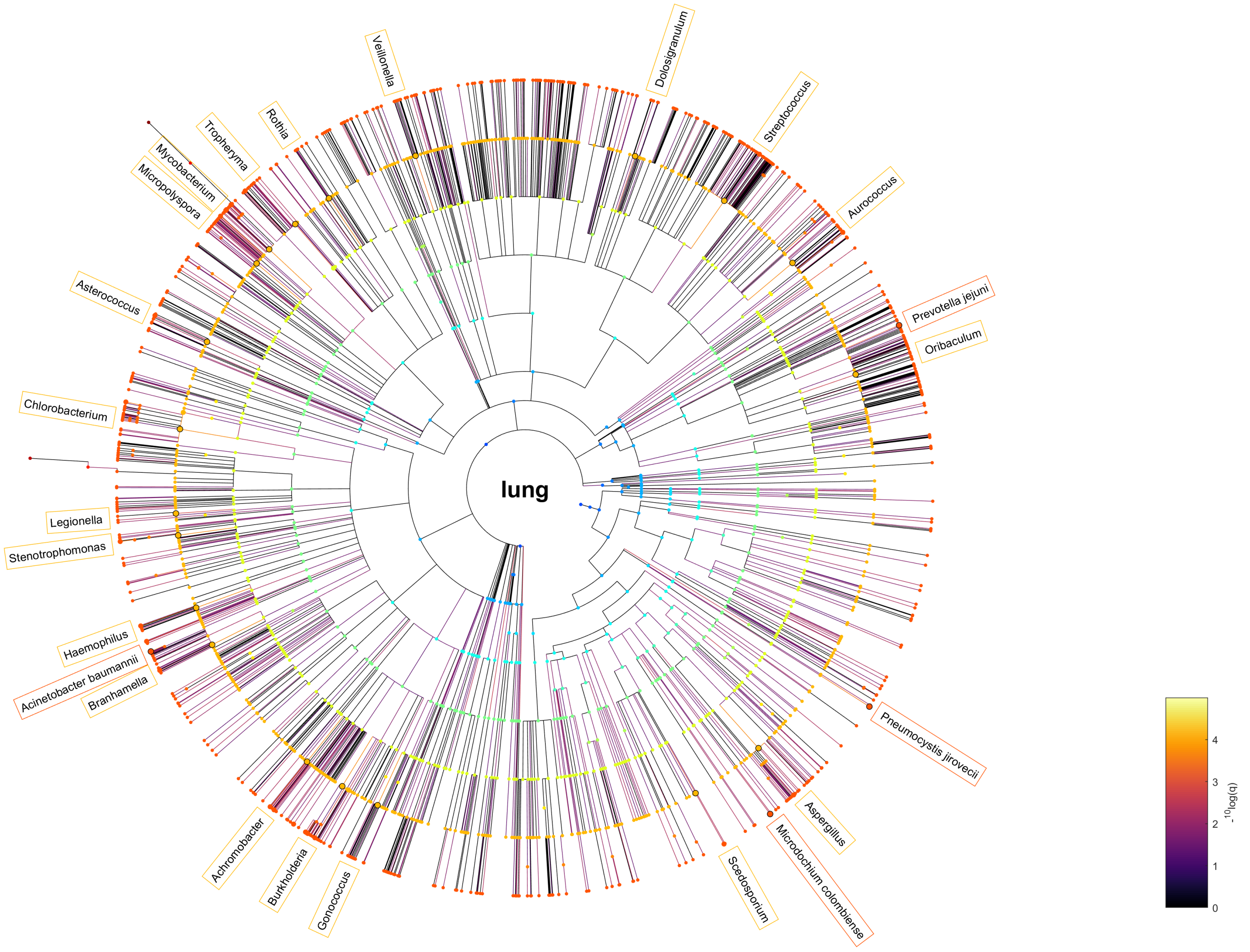

Fig. S5: Taxonomic tree visualisation of the lung (including respiratory tract) microbiota. Colour of the nodes relate to the taxonomic rank. The colour of the edges is proportional to the -log10 of the q-value (higher meaning more significant). The top microbial entities associated with the domain are visualised around the graph and coloured based on the taxonomic rank.

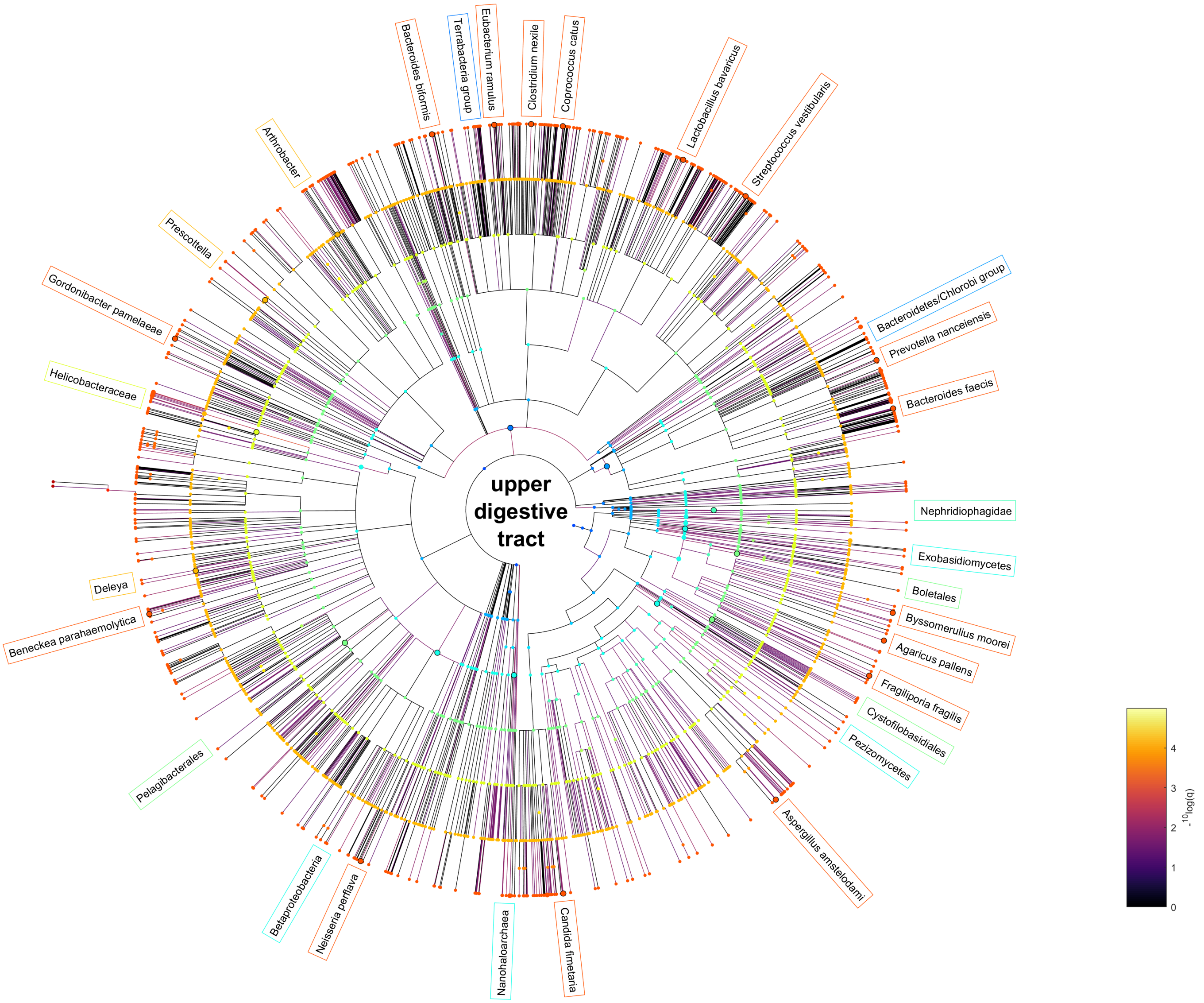

Fig. S6: Taxonomic tree visualisation of the upper gastrointestinal (oesophagus to duodenum) microbiota. Colour of the nodes relate to the taxonomic rank. The colour of the edges is proportional to the -log10 of the q-value (higher meaning more significant). The top microbial entities associated with the domain are visualised around the graph and coloured based on the taxonomic rank.

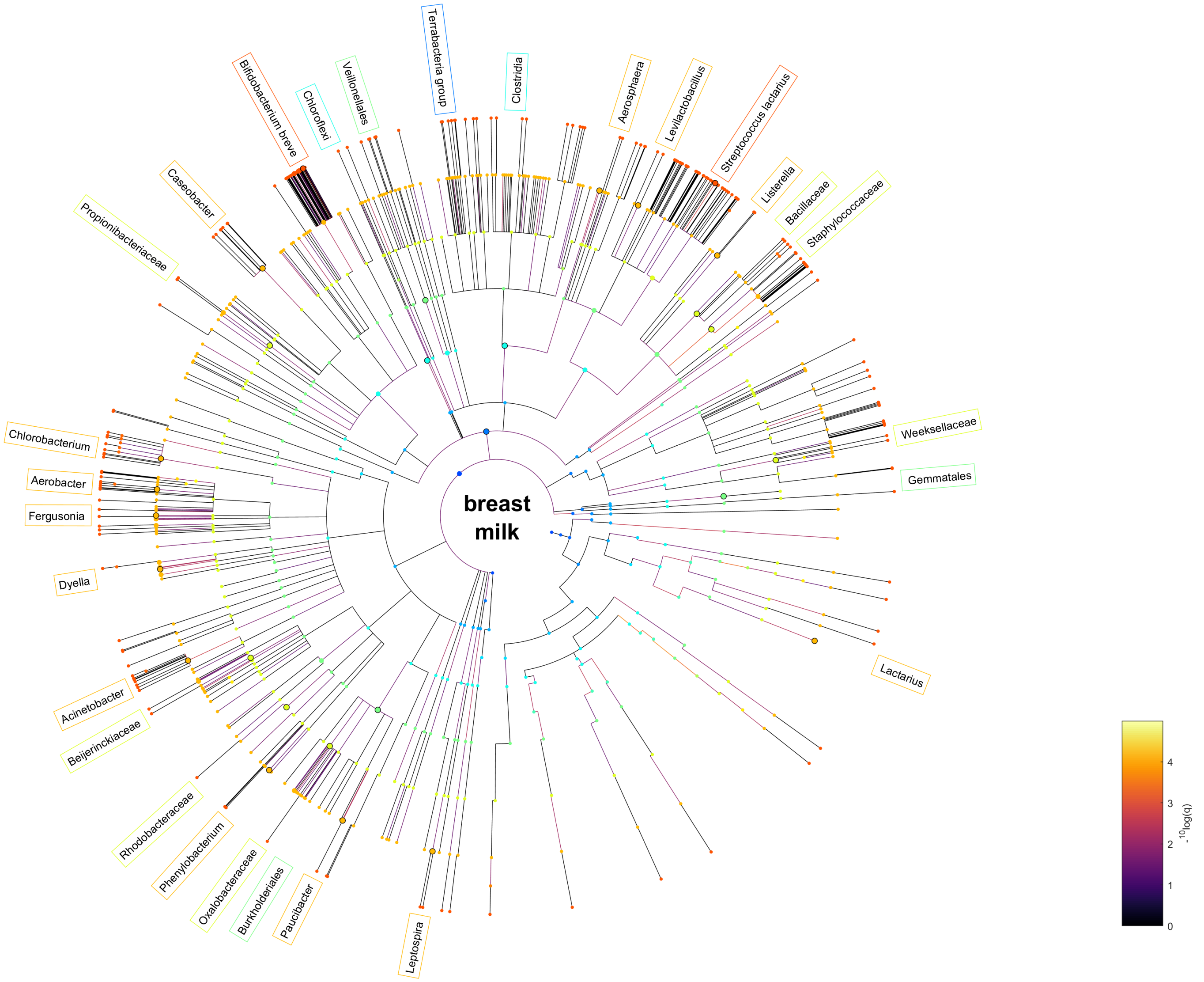

Fig. S7: Taxonomic tree visualisation of the breast milk (human milk, colostrum, lactation) microbiota. Colour of the nodes relate to the taxonomic rank. The colour of the edges is proportional to the -log10 of the q-value (higher meaning more significant). The top microbial entities associated with the domain are visualised around the graph and coloured based on the taxonomic rank.

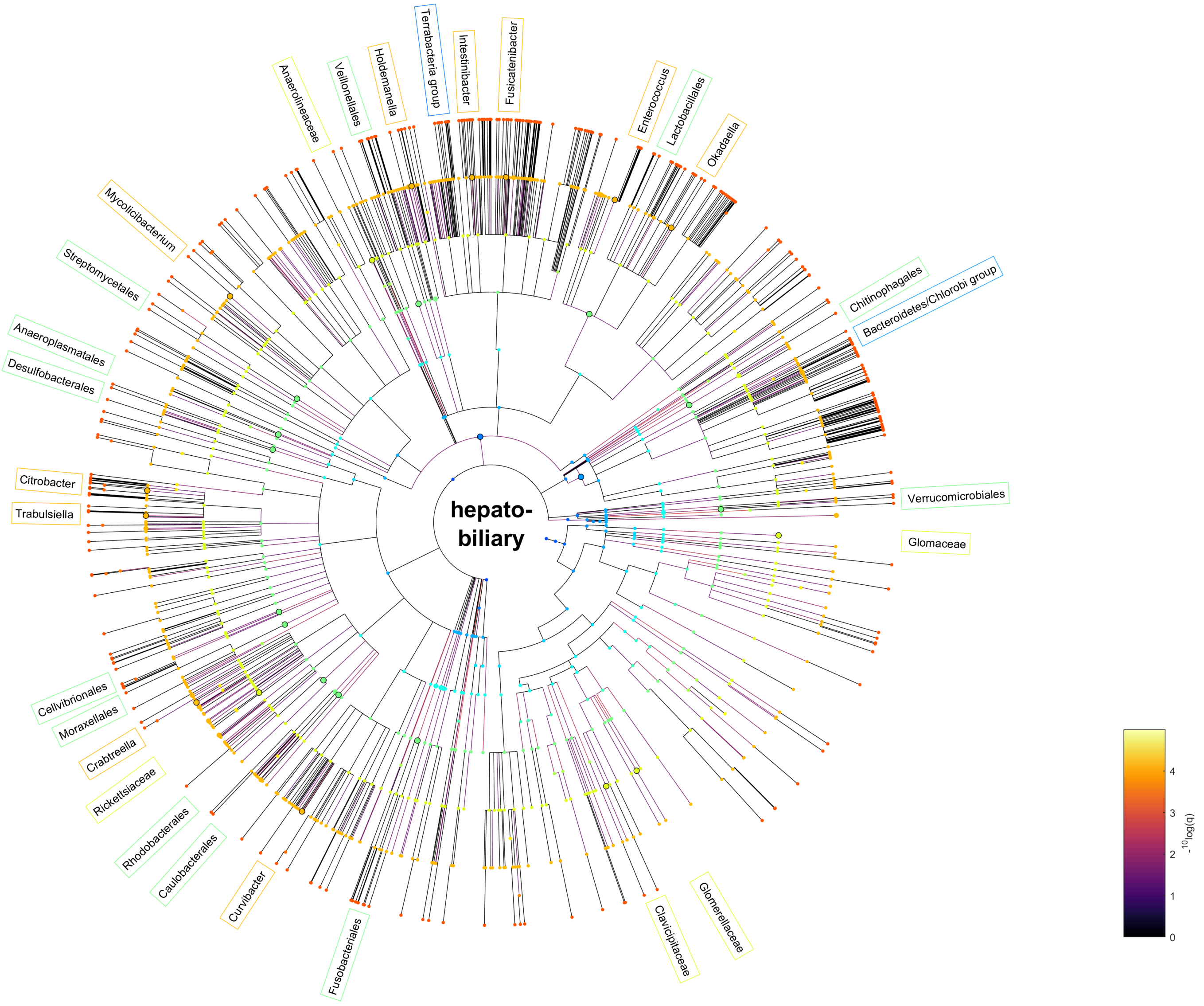

Fig. S8: Taxonomic tree visualisation of the hepatobiliary (liver, pancreas, bile duct, gall bladder) microbiota. Colour of the nodes relate to the taxonomic rank. The colour of the edges is proportional to the -log10 of the q-value (higher meaning more significant). The top microbial entities associated with the domain are visualised around the graph and coloured based on the taxonomic rank.

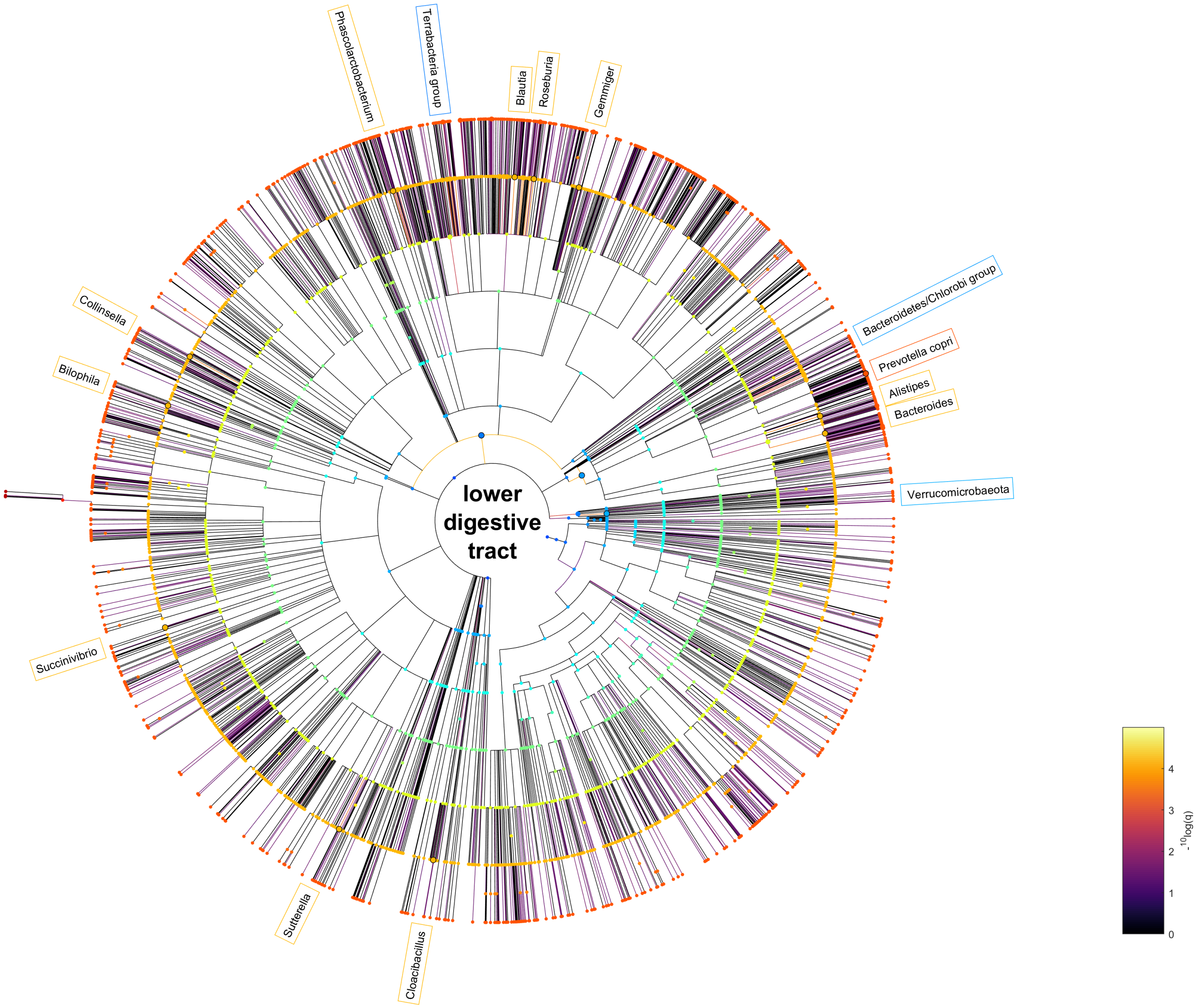

Fig. S9: Taxonomic tree visualisation of the lower gastrointestinal (jejunum to anus) microbiota. Colour of the nodes relate to the taxonomic rank. The colour of the edges is proportional to the -log10 of the q-value (higher meaning more significant). The top microbial entities associated with the domain are visualised around the graph and coloured based on the taxonomic rank.

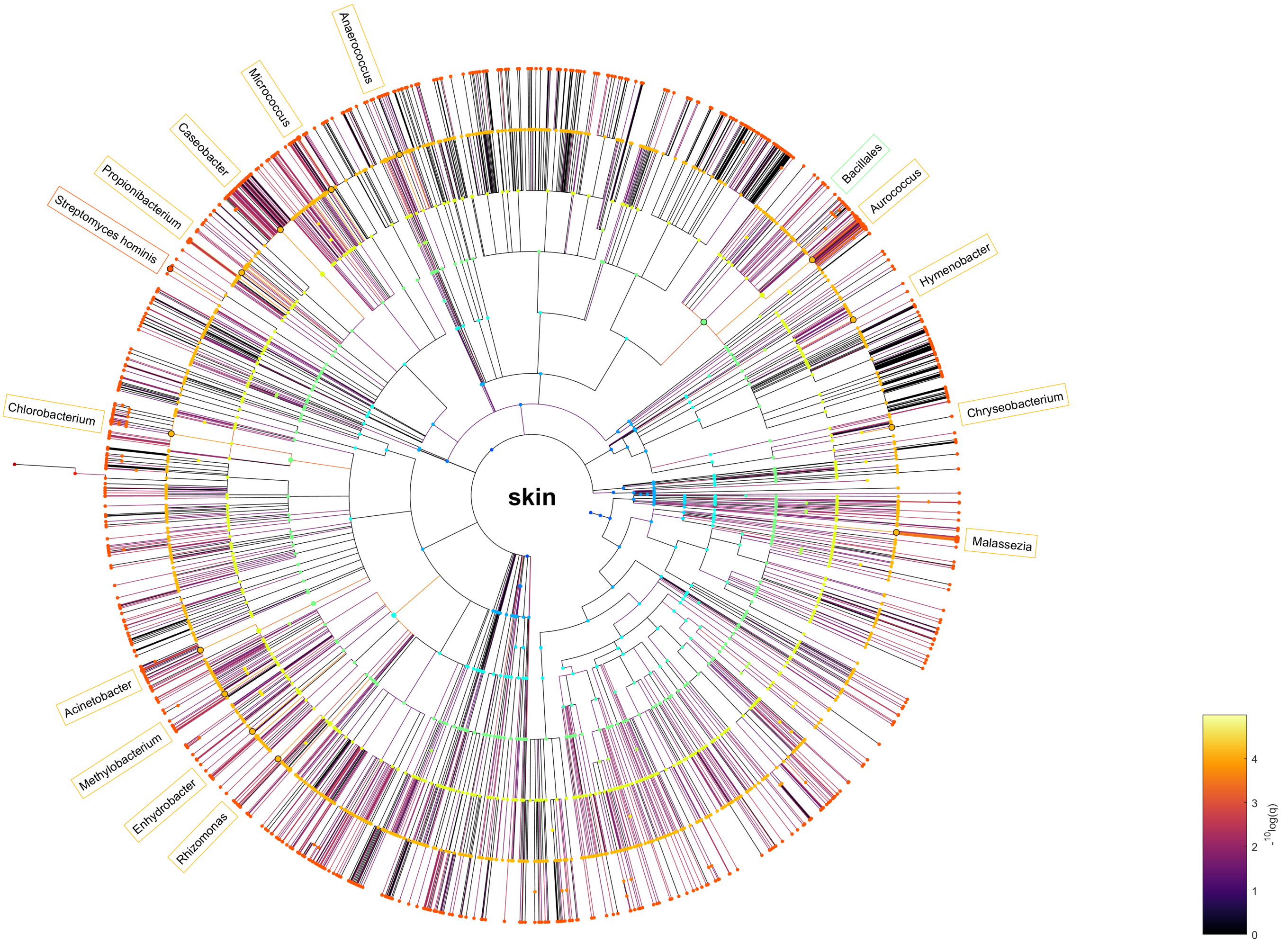

Fig. S10: Taxonomic tree visualisation of the skin (skin, toe, foot, elbow fold, forehead) microbiota. Colour of the nodes relate to the taxonomic rank. The colour of the edges is proportional to the -log10 of the q-value (higher meaning more significant). The top microbial entities associated with the domain are visualised around the graph and coloured based on the taxonomic rank.

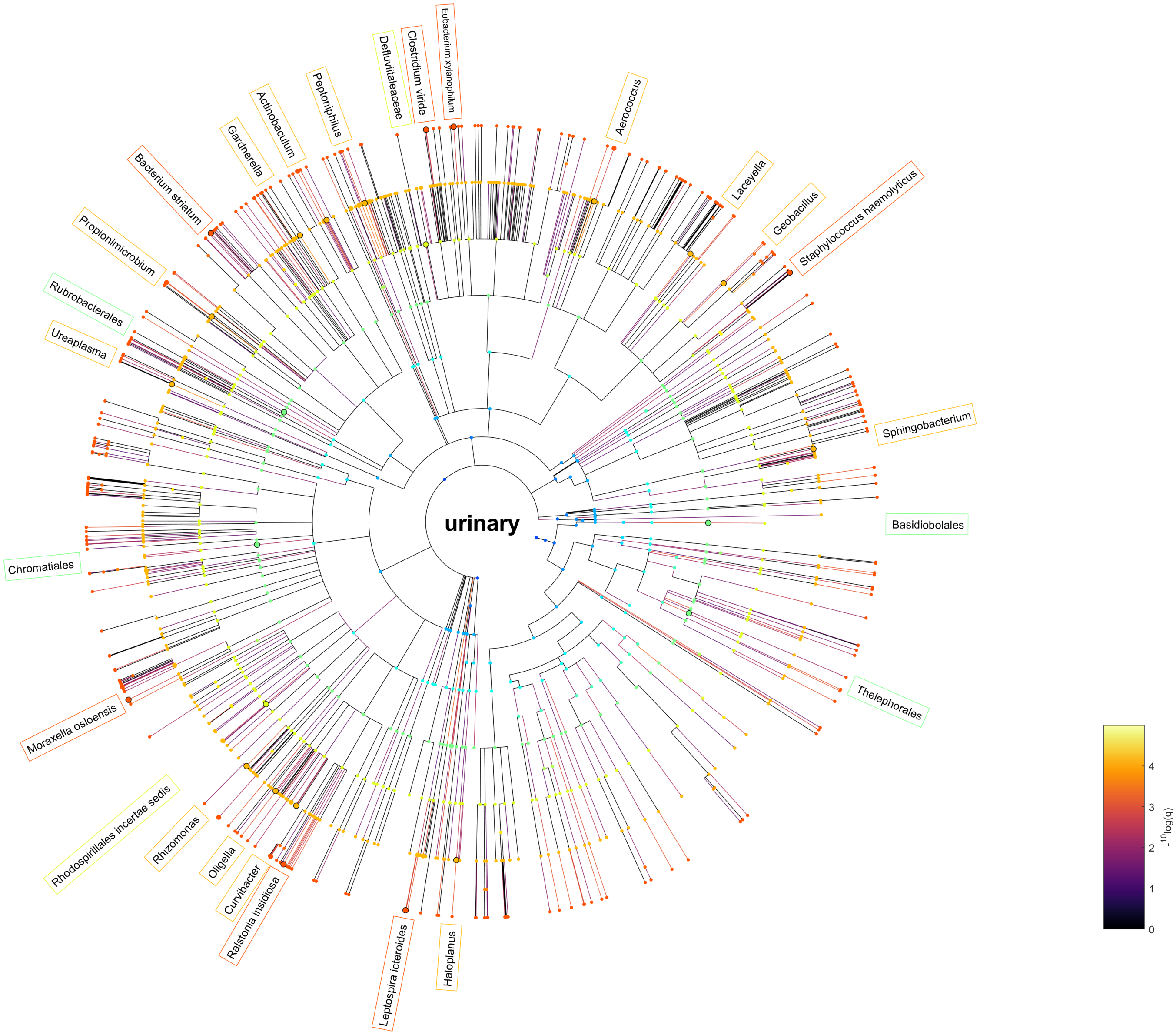

Fig. S11: Taxonomic tree visualisation of the urinary microbiota. Colour of the nodes relate to the taxonomic rank. The colour of the edges is proportional to the -log10 of the q-value (higher meaning more significant). The top microbial entities associated with the domain are visualised around the graph and coloured based on the taxonomic rank.

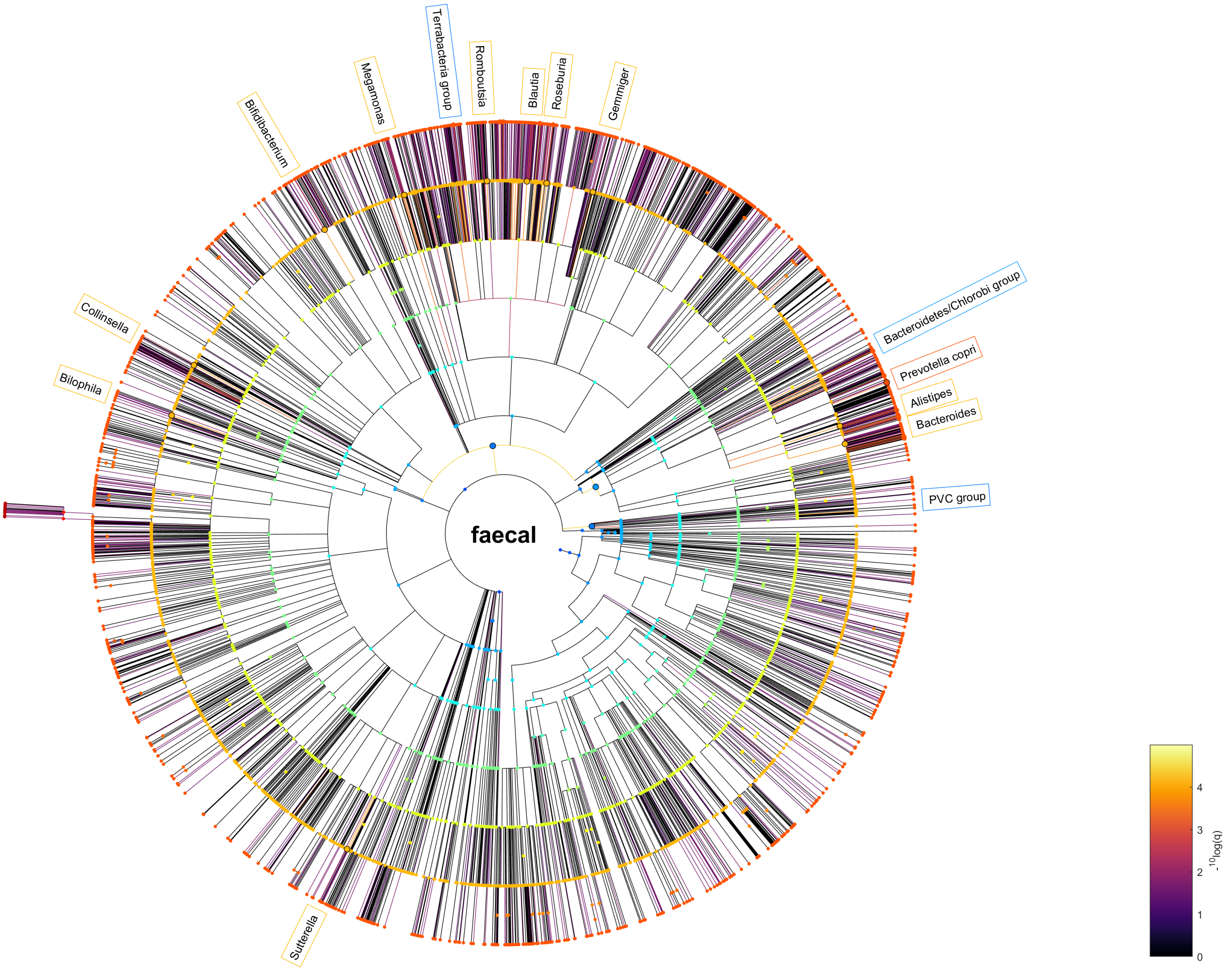

Fig. S12: Taxonomic tree visualisation of the faecal (faecal, stool) microbiota. Colour of the nodes relate to the taxonomic rank. The colour of the edges is proportional to the -log10 of the q-value (higher meaning more significant). The top microbial entities associated with the domain are visualised around the graph and coloured based on the taxonomic rank.

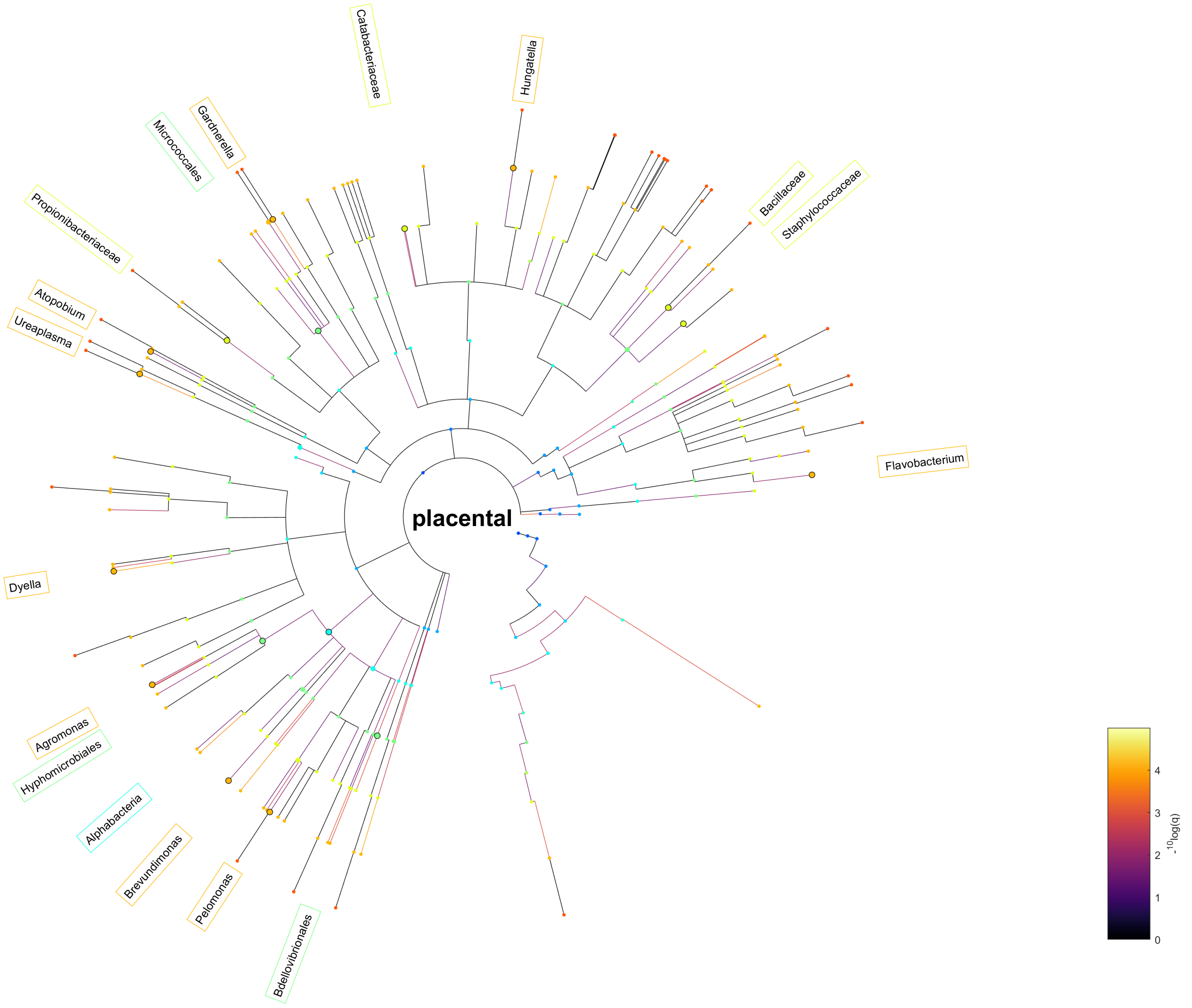

Fig. S13: Taxonomic tree visualisation of the placental microbiota. Colour of the nodes relate to the taxonomic rank. The colour of the edges is proportional to the -log10 of the q-value (higher meaning more significant). The top microbial entities associated with the domain are visualised around the graph and coloured based on the taxonomic rank.

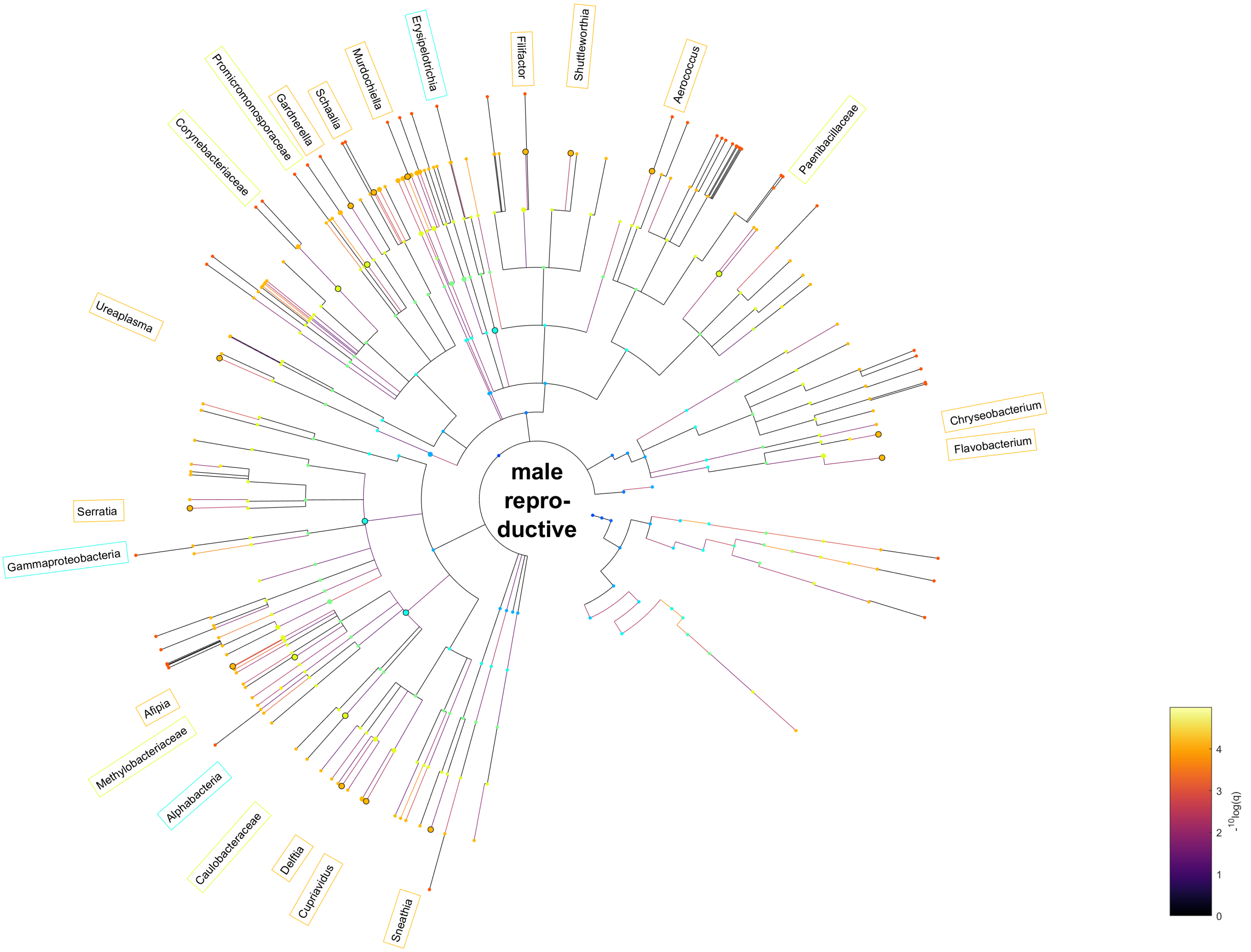

Fig. S14: Taxonomic tree visualisation of the male reproductive (testicle, semen, but not urinary) microbiota. Colour of the nodes relate to the taxonomic rank. The colour of the edges is proportional to the -log10 of the q-value (higher meaning more significant). The top microbial entities associated with the domain are visualised around the graph and coloured based on the taxonomic rank.

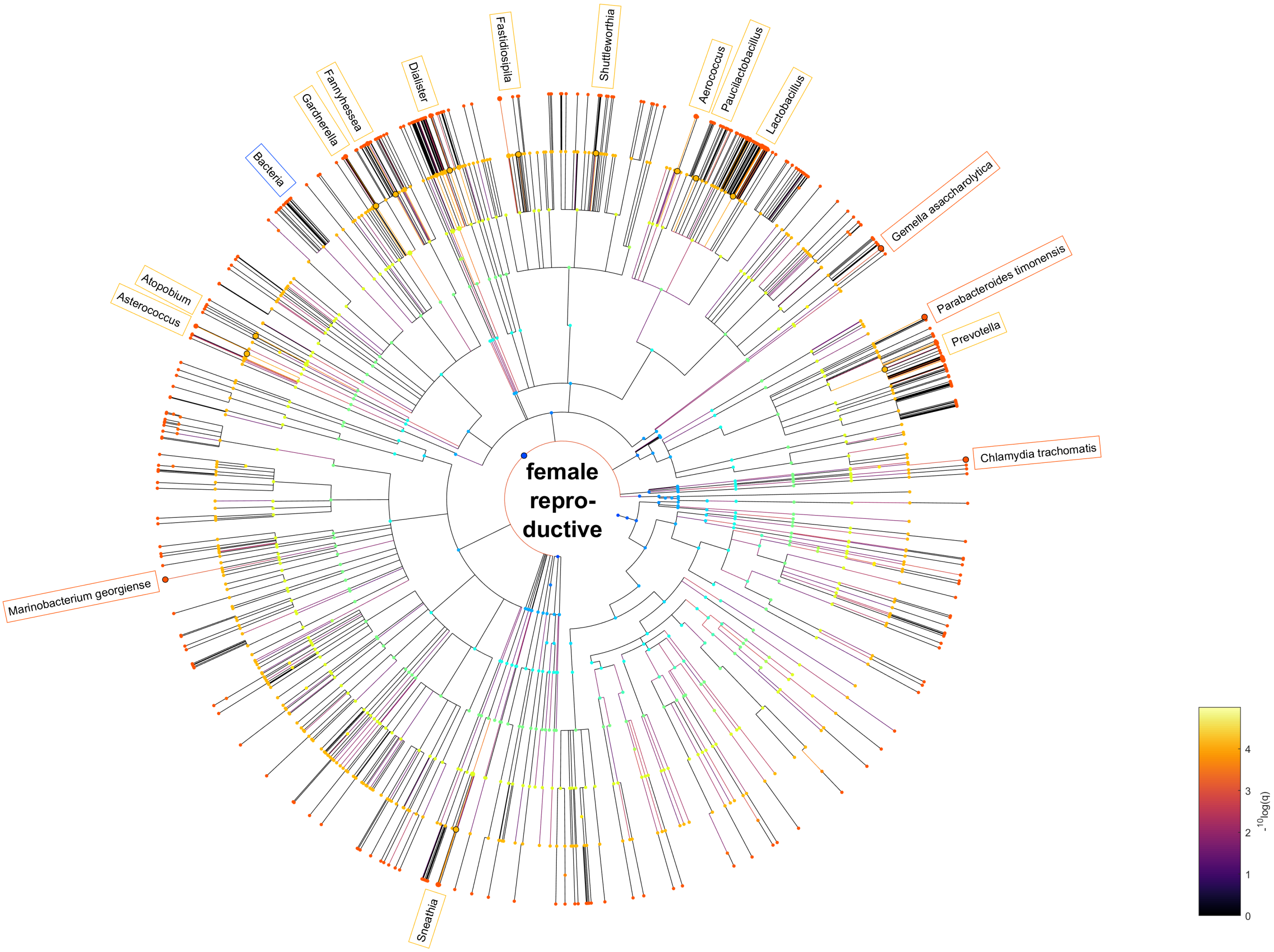

Fig. S15: Taxonomic tree visualisation of the female reproductive (vagina, cervix, endometrium, but not urinary) microbiota. Colour of the nodes relate to the taxonomic rank. The colour of the edges is proportional to the -log10 of the q-value (higher meaning more significant). The top microbial entities associated with the domain are visualised around the graph and coloured based on the taxonomic rank.
